# Correlation of LNCR rasiRNAs Expression with Heterochromatin Formation during Development of the Holocentric Insect *Spodoptera frugiperda*

**DOI:** 10.1371/journal.pone.0024746

**Published:** 2011-09-30

**Authors:** Slavica Stanojcic, Sylvie Gimenez, Emmanuelle Permal, François Cousserans, Hadi Quesneville, Philippe Fournier, Emmanuelle d'Alençon

**Affiliations:** 1 UMR1333 INRA, Université Montpellier II, Montpellier, France; 2 UR1164, INRA, Centre de Recherche de Versailles, bât.18, RD10, Versailles, France; Oregon State University, United States of America

## Abstract

Repeat-associated small interfering RNAs (rasiRNAs) are derived from various genomic repetitive elements and ensure genomic stability by silencing endogenous transposable elements. Here we describe a novel subset of 46 rasiRNAs named LNCR rasiRNAs due to their homology with one long non-coding RNA (LNCR) of *Spodoptera frugiperda*. LNCR operates as the intermediate of an unclassified transposable element (TE-LNCR). TE-LNCR is a very invasive transposable element, present in high copy numbers in the *S. frugiperda* genome. LNCR rasiRNAs are single-stranded RNAs without a prominent nucleotide motif, which are organized in two distinct, strand-specific clusters. The expression of LNCR and LNCR rasiRNAs is developmentally regulated. Formation of heterochromatin in the genomic region where three copies of the TE-LNCR are embedded was followed by chromatin immunoprecipitation (ChIP) and we observed this chromatin undergo dynamic changes during development. In summary, increased LNCR expression in certain developmental stages is followed by the appearance of a variety of LNCR rasiRNAs which appears to correlate with subsequent accumulation of a heterochromatic histone mark and silencing of the genomic region with TE-LNCR. These results support the notion that a repeat-associated small interfering RNA pathway is linked to heterochromatin formation and/or maintenance during development to establish repression of the TE-LNCR transposable element. This study provides insights into the rasiRNA silencing pathway and its role in the formation of fluctuating heterochromatin during the development of one holocentric organism.

## Introduction

Repeat associated small interfering RNAs (rasiRNAs) are described as a subset of piRNAs (Piwi-interacting RNAs), involved in defence of the eukaryotic genome against transposable elements (TEs) and viruses. RasiRNAs were first identified in *Drosophila melanogaster* and *Danio rerio*. Their lengths range from 23 to 30 nt and they are implicated in the silencing of retrotransposons in the germ line [1]–[3]. Production of rasiRNAs does not require processing of a dsRNA precursor by Dicer - a key enzyme for microRNA and siRNA production [4], [5]. In *D. melanogaster* ovaries, rasiRNAs are immunopurified with Piwi protein from heterochromatic and repetitive regions enriched in retrotransposons [6]. *Drosophila* mutants in rasiRNA pathways lead to elevated transposition incidents [7].

Most *D. melanogaster* piRNAs seem to be generated from both genomic strands (dual-strand clusters) of TE-rich regions embedded in pericentromeric and sub-telomeric heterochromatin. However, both the COM and *flamenco* loci - located in the pericentromeric heterochromatin of the X-chromosome - produce piRNAs predominantly from one genomic strand (uni-strand clusters). These piRNAs control the mobilization of retrotransposons *via* a homology-dependent gene-silencing mechanism [8]–[11]. Dual-strand piRNAs are generated by a “ping-pong” amplification cycle, while biogenesis of single-strand piRNAs cannot be assigned to ping-pong pairs [8]. Dual-strand piRNAs (rasiRNAs) reveal pairs of RNAs with complementarities in their first 10 nucleotides [12]. Two different RNA silencing pathways are active in ovarian (Piwi-dependent) *versus* other somatic tissues (Piwi independent) [11]. The Piwi-independent pathway involves endogenous siRNAs (endo-siRNAs) which are bound to AGO2 (see [13], [14], [15] for review).

piRNAs have important roles in germline development in flies, fish, and mice (reviewed in [16]). In *Drosophila* mutants, reduced abundance of rasiRNAs derived from a wide range of TEs is accompanied by an increase of euchromatic markers (H3K4me2) and a decrease of heterochromatic markers (decrease of H3K9me2/3 and depletion of HP1 content) within the chromatin of retrotransposons [7]. Furthermore, short interfering RNAs are implicated in chromatin modifications, such as H3K9me2, in yeast, plants and animal somatic cells [17]–[20].

*Spodoptera frugiperda*, also known as “fall armyworm”, belongs to the insect order Lepidoptera, as does the domesticated silkworm (*Bombyx mori*), the recognized lepidopteran model insect. *S. frugiperda* is one of the most destructive pests of agricultural crops in the world and it is mainly studied for pesticide resistance, midgut enzymes and host-virus interactions. As other Lepidoptera, it is a holometabolic insect whose development starts from eggs, followed by 6 larval stages (L1 to L6) and a pupal stage prior to becoming adult. The *S. frugiperda* genome is organised into 31 chromosome pairs with an estimated size of 400 Mb [21]. Only a partial genomic sequence of *S. frugiperda* is available (4.67 Mb *i.e.* 1% of the genome cloned in 37 BACs) [22]. The genomic regions cloned in BACs are 101 to 205 Kb long and they represent scattered genomic regions from different chromosomes [22]. Like other Lepidoptera, Hemiptera, and Heteroptera, *S. frugiperda* is a holocentric species, with diffuse kinetochore along the entire length of the mitotic chromosomes [21].

Here we describe a novel subset of 46 rasiRNAs from *S. frugiperda*, nominated LNCR rasiRNAs due to homology with one long non-coding RNA (LNCR) which operates as intermediate of an unclassified transposable element (TE-LNCR). LNCR rasiRNAs are single-stranded RNAs, unidirectional and organized into two distinct clusters. The expression of LNCR rasiRNAs, as well as their parental LNCR, is developmentally regulated. All LNCR rasiRNAs expressed in eggs and L2 larvae are 36 nt long while in the pupae most are 33 nt long. They do not have prominent nucleotide motifs and their expression pattern is qualitatively and quantitatively altered at different developmental stages. The higher expression of LNCR in certain developmental stages is followed by the generation of an assortment of LNCR rasiRNAs which appears to correlate with the presence of a heterochromatic histone mark in genomic regions enriched with inserted copies of the TE-LNCR. The potential role of LNCR rasiRNAs in dynamic heterochromatin formation and development is discussed.

## Results

### Deep sequencing of small RNAs and annotation of 102 rasiRNAs

**Figure 1:**
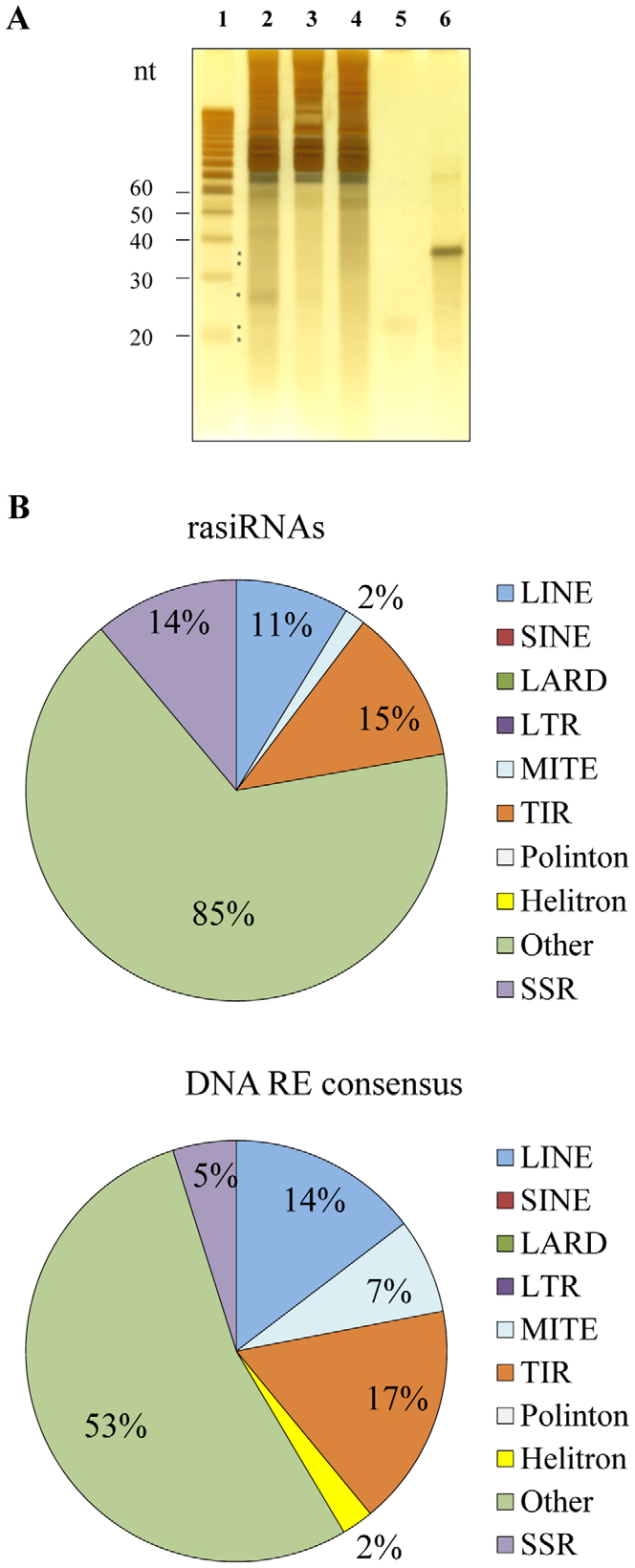
Distribution of small RNAs in three developmental stages of *S. frugiperda* and annotation of mapped rasiRNAs. A) Total RNAs (5 µg) from three developmental stages of *S. frugiperda* were loaded onto a 17% polyacrylamide gel containing 7 M urea and stained with silver after electrophoretic separation. Molecular sizes (in nucleotides) are shown on the left of the figure. Some discrete bands of small RNAs are labeled with asterisks. 1) Decade small RNA ladder, 2) total RNA 2.5 days old fertilized eggs. 3) total RNA L2 larvae, 4) total RNA 12 days old pupae, 5) small synthetic RNA 21 nt, 6) small synthetic RNA 36 nt. B) Pie charts summarizing the annotation of 102 rasiRNAs, mapped on consensus DNA repeated elements (upper panel) and the annotation of consensus DNA repeated elements defined after analysis of 1% of the sequenced *S. frugiperda* genome (lower panel).

Total RNA was prepared from the whole *Spodoptera frugiperda* body at three developmental stages; 2.5 days old fertilized eggs, L2 larval stage, and 12 days old pupae. The quality and quantity of total RNA was checked on an agarose gel stained with EtBr (**Figure S1**) and the quality and quantity of small RNA was checked on polyacrylamide denaturing gels stained with silver (**Figure 1A** **and Figure S1B**). A few distinct bands of small RNAs in the range of 15–40 nucleotides were observed (labeled with asterisk in **Figure 1A**). One of the most prominent bands of small RNA is the one around 27–28 nt, already described as piRNA in *Bombyx mori* and *S. frugiperda* cell lines [23]. Small RNAs libraries from three developmental stages were prepared (see Materials and Methods) and sequenced on an Illumina 1G genome analyzer. The numbers of sequences obtained at each developmental stage are presented in **Table S1**. After removal of low-quality reads, homopolymers longer than 12 nt, and sequences shorter than 10 nt, 5,405,284 reads of small RNA were obtained in eggs, 5,678,498 in L2 larval stage and 11,167,633 reads in pupae. Of the 1,595,008 unique small ncRNA sequences present in all three libraries, 12.3% matched perfectly with *S. frugiperda* rDNA while 2.3% were similar to *D. melanogaster* tRNA, indicating a rather low rate of RNA degradation.

In this study we specifically focused on rasiRNA of *S. frugiperda*. Of the 1,595,008 unique small ncRNA sequences present in all three libraries, only 102 different rasiRNAs longer than 15 nt, were annotated. The low number of annotated rasiRNAs is caused by the lack of complete *S. frugiperda* genomic data (only 1% of sequenced genome and consequently - incomplete identification of DNA repeated elements). The annotated rasiRNAs match perfectly to previously defined DNA repeated elements (DNA RE), identified from the analysis of sequenced genomic regions cloned in 37 BACs using the REPET pipelines [22], [24]. The annotation of 102 rasiRNAs (only 0.006% of all unique small RNA sequences of the three libraries) is shown in the upper graph of **Figure 1B**. Most rasiRNAs were mapped onto unclassified DNA RE (85%), indicated as other in **Figure 1B**. Unclassified DNA REs have no structural elements allowing their classification either as retrotransposons or DNA transposons. 17% of rasiRNAs map to the identified DNA transposons (15% to terminal inverted repeats (TIRs) and 2% to miniature inverted-repeat transposable elements (MITEs)). 11% of rasiRNAs map to retrotransposon of the long interspersed nuclear elements (LINEs). In addition, 14% of rasiRNAs map to one simple sequence repeats (SSR) consensus. A similar distribution of DNA RE consensus sequences was observed in the sequenced genomic portion of *S. frugiperda* (**Figure 1B**, lower graph). These DNA RE consensus sequences were characterized using the REPET pipelines [22], [24], and it was observed that 1% of the *S. frugiperda* genome contains mostly unclassified (indicated as other in graph) repetitive elements (53%), and then 17% TIR, 14% LINE, 7% of MITE, and 5% of SSR as DNA repetitive elements.

We anticipate that the whole genome sequencing and sequence composition analysis will bring new insights into all of the DNA repeated sequences present in the *S. frugiperda* genome, making the complete annotation of all rasiRNAs possible.

### A novel subset of *S. frugiperda* rasiRNAs associated with one long non coding RNA (LNCR rasiRNAs)

In this study we further characterized a novel subset of 46 rasiRNAs representing most of the 102 annotated *S. frugiperda* rasiRNAs (45% of all annotated rasiRNAs). All of these 46 rasiRNAs mapped to one consensus sequence of unclassified DNA repeated element, initially named Spodo2-B-R19-Map11_NoCat [22]. The consensus sequence is made from alignment of 11 copies of DNA repeated elements identified within 1% of *S. frugiperda* genome by the REPET pipelines [22], [24]. This consensus sequence is 2007 bp long and devoid of structural elements allowing its classification as a retrotransposon or a DNA transposon, except short tandem repeats (STR) at one end. STRs have been already described as structural elements in some retrotransposons [25]. We estimated that there are between 1600 and 2050 copies of TE-LNCR in the *S. frugiperda* genome by relative qPCR (relative TE-LNCR copy number is determined by comparison with ribosomal gene L37, data not shown) and we found on average a copy every 257 kb in the available genomic sequence. At the same time, the analysis of *S. frugiperda* high-throughput sequenced RNA revealed a 1443 nt long RNA sequence (contig sequence derived from 84 overlapping reads obtained with 454/Roche sequencing of total RNA expressed in all developmental stages) which included all 46 rasiRNA sequences. Six-frame translation of this long RNA did not generate any protein of significant similarity with already characterised proteins. It may therefore correspond to a long non-coding RNA (LNCR). Consequently the 46 rasiRNAs are named LNCR associated rasiRNAs (LNCR rasiRNAs) and the repeated element was named TE-LNCR (see **Table S2** for accession numbers of 46 LNCR rasiRNAs, 11 TE LNCR copies and LNCR).

**Figure 2:**
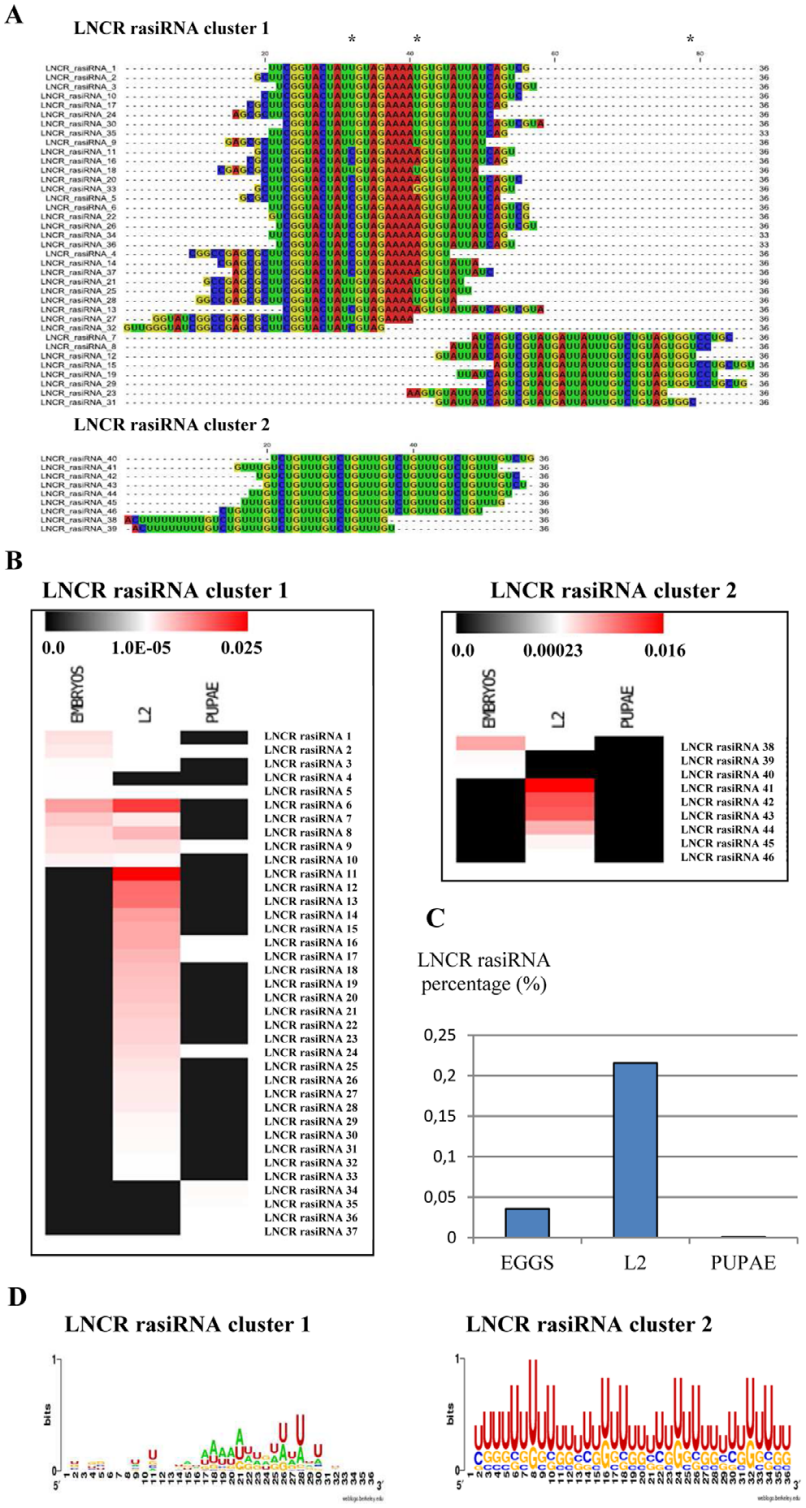
LNCR rasiRNAs expression during different developmental stages of *S. frugiperda*. A) Alignment of LNCR rasiRNA sequences organised in two clusters by CLC Sequence Viewer 6.3 software. All the LNCR rasiRNA sequences are acquired from Illumina sequencing. Asterisks are pointing imperfect matches. B) Expression heat map of LNCR rasiRNAs acquired by Illumina sequencing of three *S. frugiperda* libraries (eggs, L2 and pupae). The normalized copy number of each LNCR rasiRNA sequence is presented using color bar at the top of each image which presents scale of percentage of LNCR rasiRNAs sequenced copies over the total number of sequenced small RNAs in each library (see Table S3 for the exact numbers). The LNCR rasiRNA abundance is positively correlated with the red color intensity. C) Percentage of total LNCR rasiRNA sequenced copies found at each developmental stage of *S. frugiperda* over the total number of sequenced small RNAs in each library acquired from Illumina sequencing of three developmental libraries (see also Table S3). D) A nucleotide composition of *S. frugiperda* LNCR rasiRNAs from both clusters (weblogo.berkeley.edu).

The sequences of all LNCR rasiRNAs, their presence at each developmental stage and their detailed positions on LNCR or unclassified consensus DNA-repeated element are presented in **Table S3**. The alignment of 46 LNCR rasiRNAs demonstrated a presence of highly overlapping sequences within the two clusters (**Figure 2A**). Cluster 1 is made of 37 LNCR rasiRNAs whereas 9 LNCR rasiRNAs make cluster 2. They are all 36 nt long, except for three 33 nt LNCR rasiRNAs from cluster 1. The sequence and alignment of LNCR and LNCR rasiRNAs are presented in **Figure S2**. LNCR rasiRNAs from cluster 1 overlap over a region of 87 nucleotides, whereas cluster 2 rasiRNAs are distributed over a region of 66 nucleotides of LNCR which encompasses short tandem repeats (seven times repeated UGUCUGUU motif on LNCR). Besides the homology to unclassified consensus DNA RE sequence mentioned above, LNCR rasiRNAs from cluster 2 are also homologous to one SSR and one TIR class II consensus sequence. Some LNCR rasiRNAs do not have perfect homology with the LNCR (one nucleotide mismatch). This is either due to individual polymorphisms or that LNCR transcription occurs at several distinct genomic sites. LNCR rasiRNAs are in the same orientation (unidirectional) and they map onto one strand of the unclassified DNA repeated element and LNCR (uni-strand clusters).

The occurrence of LNCR rasiRNAs during the different developmental stages of *S. frugiperda* is variable (**Figure 2B** and **Table S3**). Some LNCR rasiRNAs are expressed predominantly in eggs (LNCR rasiRNAs #1 to #5 from cluster 1, LNCR rasiRNAs #38 to #40 from cluster 2). Other LNCR rasiRNAs are expressed both in eggs and L2 larval stage (LNCR rasiRNAs #6 to #10 from cluster 1), whereas a large number of LNCR rasiRNAs are expressed exclusively in the L2 stage (LNCR rasiRNAs #11 to #33 from cluster 1, LNCR rasiRNAs #41 to #46 from cluster 2). Finally, LNCR rasiRNAs #34 to #37 from cluster 1 are solely expressed in the pupal stage. The length of LNCR rasiRNAs specific to the pupal developmental stage is 33 nt; the others are 36 nt long. The variety and quantity of expressed LNCR rasiRNAs was highest in the L2 larval stage (0.22% of all high quality sequenced small RNAs detected in L2 larval stage library), followed by the eggs (0.036% of all high quality sequenced small RNAs detected in eggs library) and then pupal stages (0.001% of all high quality sequenced small RNAs detected in pupal library) (**Figure 2C** and **Table S3**).

The nucleotide composition of both LNCR rasiRNA clusters is presented as **Figure 2D**. LNCR rasiRNAs from cluster 2 are poor in adenine and very rich in uridine (63%). No bias at the 5′ end of sequences was observed, but a small bias towards uridine at position 28 in cluster 1 (57%) was detected. Furthermore, a bias towards uridine at position 8 (77%) in cluster 2 was detected that may be caused by the fact that it is made of Short Tandem Repeats (STR). The most probable secondary structures of *S. frugiperda* LNCR rasiRNAs are shown in **Figure S3**. LNCR rasiRNAs from cluster 2 display very similar secondary structures with one small loop close to the 5′ end. LNCR rasiRNAs from cluster 1 show more variable secondary structures, but most have one hairpin structure with double stranded RNA and loops of various sizes.

### Long ncRNA (LNCR) expression is developmentally regulated

**Figure 3:**
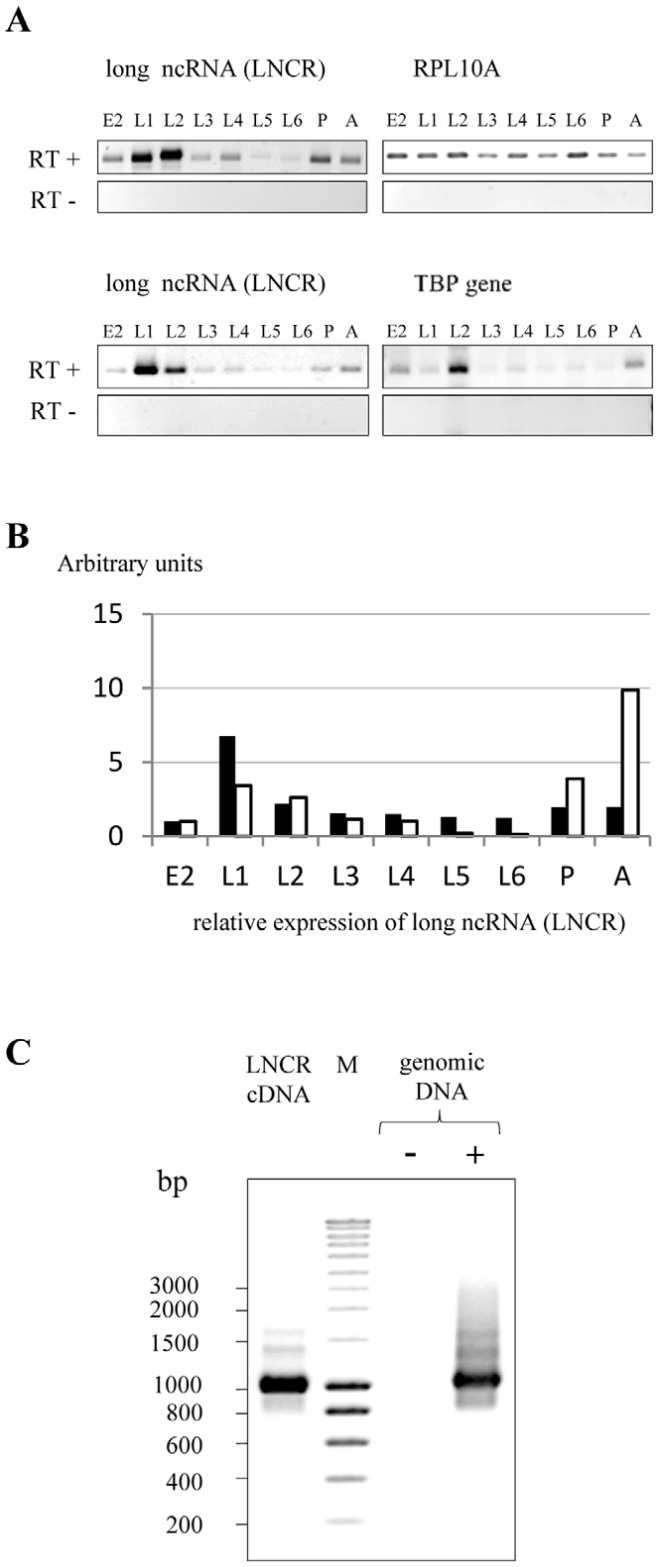
Expression analysis of *S. frugiperda* long ncRNA (LNCR) during different developmental stages. A) Expression analysis of *S. frugiperda* long ncRNA (LNCR) during different developmental stages (E2- 2.5 days old fertilised eggs, L1–L6 larval stages, P- 12-day old pupae and A- adults) by semi quantitative RT PCR. PCR was done on total RNA treated with reverse transcriptase (RT+) or without it (RT−). Expressions of ribosomal gene RPL10A and TBP (TATA binding protein) were used as endogenous control genes. PCR was done with primers amplifying the LNCR region from 27–1271 (upper panel) and LNCR region from 858–1271 nt (lower panel). B) Graph presenting the normalized quantity of expressed LNCR relative to the expression of endogenous control genes: ribosomal gene RPL10A (white bars) and transcription factor TBP (black bars). The quantification was done with ImageQuant TL software. C) PCR done on *S. frugiperda* genomic DNA with the same pair of primers (pair of primers amplifying the LNCR region from 27–1271 nt) used for expression analysis of LNCR by semi quantitative RT PCR. M indicates DNA ladder and molecular sizes (in base pairs) are shown on the left of the figure.

To confirm expression of the LNCR, a semi quantitative RT-PCR analysis was performed on total RNA expressed at different developmental stages of *S. frugiperda*. Two pairs of primers, designed at different regions of LNCR, were used for this analysis (see Materials and Methods). A band of the expected size was obtained with each pair of primers, and no band was detected in absence of RT (**Figure 3A**). This implies that the LNCR is expressed in all developmental stages *in vivo*. LNCR expression was variable during the different developmental stages compared with the expression of two house-keeping genes used as controls (ribosomal protein RPL10A and TATA binding protein - TBP). The quantification and normalisation of semi quantitative RT-PCR is shown on **Figure 3B**. In addition, dynamic transcription analysis of LNCR at different developmental stages was performed also by qRT-PCR with two pairs of primer (LNCR 1 and LNCR 2, see Material and Methods) and the relative copy number of LNCR was determined in comparison with expression of ribosomal gene RPL37 as endogenous control (**Figure S4**). In summary, both methods show that LNCR expression displays a peak at the L1 larval stage and then decreases until the pupal and adult stage, where the expression of LNCR is increased again. Expression of LNCR is therefore developmentally regulated.

LNCR is the intermediate of a very invasive transposable element (TE-LNCR), with an estimated copy-number of 1600 to 2050 per genome. The exact positions of LNCR rasiRNAs associated to TE-LNCR copies were mapped onto some of the already sequenced BACs and presented in **Table S4**. The inserted genomic copies of TE-LNCR are occasionally degenerated or rearranged, with a variable conservation rate. Within the 1% of sequenced genome, we did not find any perfect match with the sequence of LNCR obtained with 454/Roche sequencing, indicating that it is transcribed from still unannotated region(s) of the genome. Using PCR analysis on genomic DNA as a template, a band of the same size as in RT PCR was obtained (**Figure 3C**). This indicates that the genomic locus/loci where transcription of LNCR occurs, does/do not contain large introns, at least in the analysed region of 1244 bp. Since it is transcribed and does not encode proteins, TE-LNCR could be a kind of non-autonomous retrotransposon, or it could be transcribed by read-through transcription from an external promoter.

**Figure 4:**
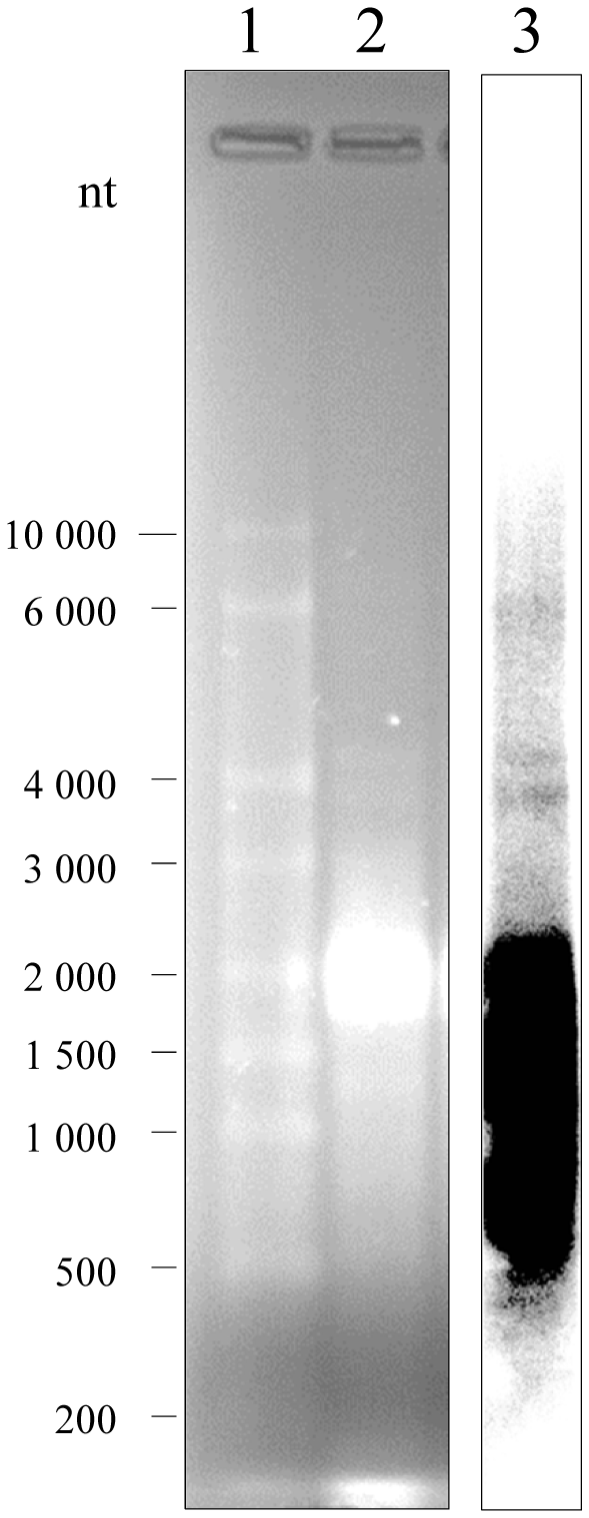
Northern blot analysis of LNCR expression in *S. frugiperda* adults. Total RNA (15 micrograms) extracted from *S. frugiperda* adults was denatured, separated on 1.2% agarose gel (lane 2), together with RNA ladder (lane 1) and stained with EtBr. Molecular sizes of RNA ladder (in nucleotides) are indicated on the left of the figure. The RNA was blotted onto nylon membrane and hybridized with a ^32^P-labeled LNCR specific DNA probe. The probe (1244 bp) was generated with ncRNA_long primers (see Material and Methods) by PCR, using cDNA of adults as template. Northern blot is shown in lane 3.

The sequence of LNCR obtained with 454/Roche sequencing is devoid of any polyA tail, which did not allow determination of its orientation and actual size *in vivo*. To determine the size of expressed LNCR transcripts *in vivo* we performed Northern blot analysis on total RNA expressed in adults (**Figure 4**). The total RNA expressed in adults was denatured and separated on 1.2% agarose gel, together with RNA ladder (lane 2 and 1, respectively in **Figure 4**). The DNA probe of 1244 bp was generated by PCR (ncRNA long primers, see Material and Methods) on adult cDNA as a template and random primed labelled with radioactive ^32^PdCTP. The Northern blot hybridisation, followed by stringent washes, is performed and the result is presented in lane 3, **Figure 4**. As we suspected, we got a smear of signals mainly in the range of 0.5 to 2 Kb, indicating that the LNCR transcription is probably starting from multiple genomic sites and that is under control of various external promoters. Multiple genomic transcription sites could be also combined with various processing ways of LNCR since it generates LNCR rasiRNAs.

### LNCR rasiRNAs and heterochromatin formation

Small ncRNAs have been shown to be involved in the silencing of transposable elements as well as in heterochromatin formation [6], [7], [20], [26]. Since the development process involves extensive epigenetic changes including histone H3 modifications, we investigated in this study the general heterochromatin and euchromatin status of the *S. frugiperda* genome during the different developmental stages. The objective was to see if there is evident accumulation of euchromatic or heterochromatic markers in certain developmental stages. To do this, the overall presence of epigenetic marks that are universally associated to heterochromatin (dimethylation of histone H3 at lysine 9 - H3K9me2) and euchromatin (dimethylation of histone H3 at lysine 4 - H3K4me2) was followed by Western-blot during different developmental stages of *S. frugiperda* (**Figure S5A**). Western-blot signals were depicted at the linear range of exposition and the quantification analysis (see Materials and Methods) of these two histone H3 modifications relative to the overall amount of histone H3 is presented in **Figure S5B**. Using this method, a statistically significant enrichment of heterochromatic mark H3K9me2 was not observed during any developmental stage, whereas the euchromatic mark H3K4me2 is slightly more represented at very early stages of embryos development.

**Figure 5:**
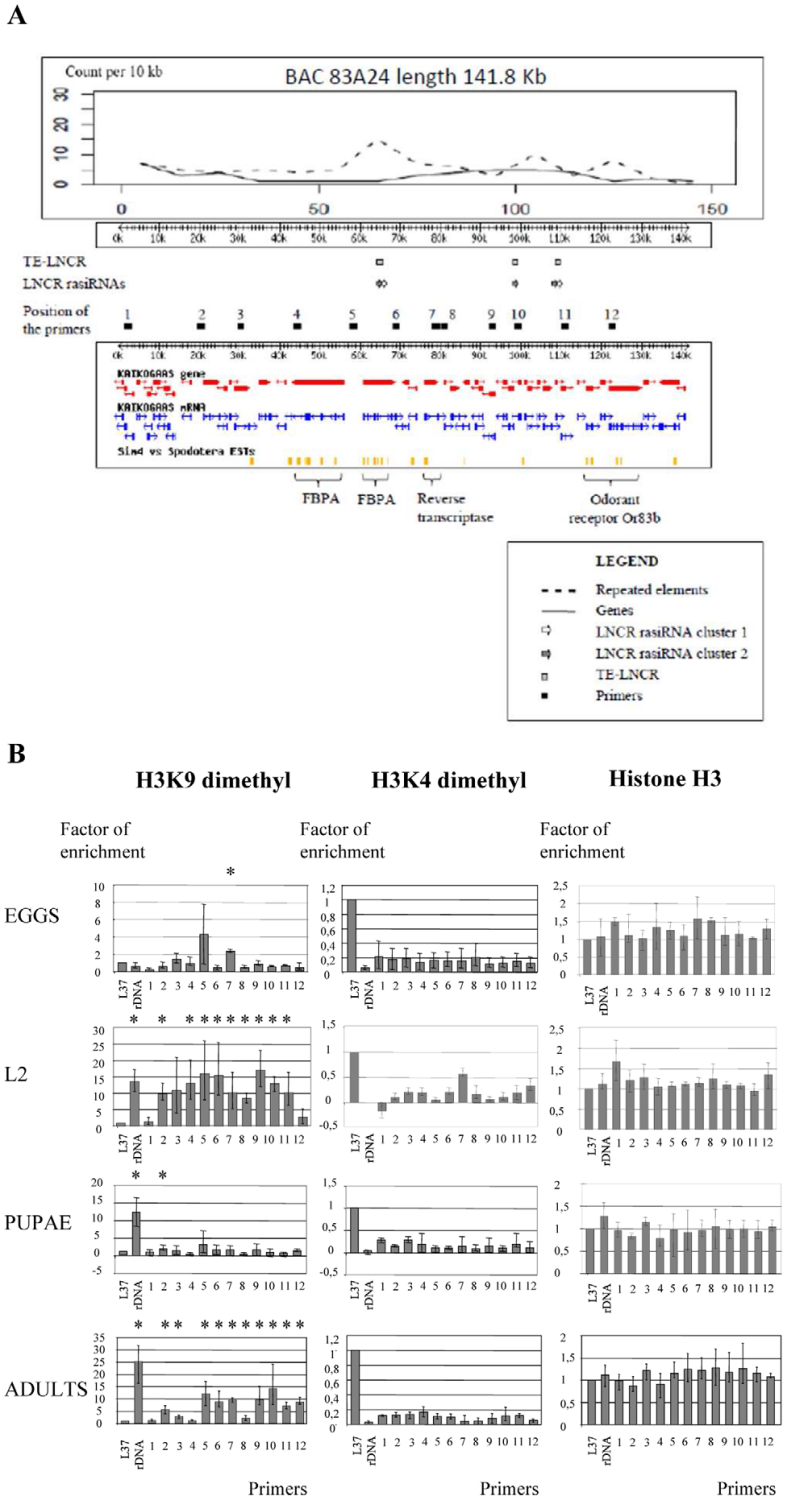
Distribution of genes, repeated elements, euchromatic and heterochromatic markers in genomic region cloned in BAC 83A24. A) Schematic presentation of genetic and epigenetic elements in the genomic region cloned in BAC 83A24 (141.8 Kb). Upper panel presents distribution of genes and DNA repeated elements in genomic region cloned in BAC 83A24. The positions of TE-LNCR, LNCR rasiRNAs and primers used in ChIP qPCR are indicated in the middle. The lower panel of the figure presents this genomic region with the position of predicted genes and mRNAs, as well as the position of *S. frugiperda* ESTs (taken from *Lepido*DB (http://genouest.org/cgi-bin/gbrowse/gbrowse/lepido_pub/)). The positions of putative genes are indicated below (FBPA gene is duplicated). B) Presence of histone H3 and histone H3 modification (H3K9me2 and H3K4me2) in analyzed genomic regions (BAC 83A24) during different developmental stages done by ChIP-qPCR. L37 ribosomal gene was used as a positive control for euchromatic regions and rDNA as a positive control of heterochromatic regions. Error bars indicate standard deviations over at least three independent experiments. Asterisks indicate statistically significant enrichment of heterochromatic marker over the control region L37 (p<0,05). P-values were calculated using Student's t-test.

To assess the kinetics of the epigenetic changes associated with *S. frugiperda* development, chromatin immunoprecipitations (ChIP) experiments were performed with antibodies that specifically recognise the above mentioned modifications of histone H3. The analyzed genomic region was a 141.1 Kb segment, cloned in BAC 83A24 (see LepidoDB, [22]). This genomic region contains three distinct insertions of the TE-LNCR (**Figure 5A**). The first one is at position 64563–65884 bp, spanning the fourth intron of the fructose 1–6 biphosphate aldolase (FBPA) gene. At this position, TE-LNCR could be transcribed from the promoter of the FBPA gene and could presumably generate all of these LNCR rasiRNAs. The other two insertions are in intergenic regions – at 98101–98697 bp (a considerably rearranged and shortened copy) and at 109439–110827 bp. These two areas are under no apparent promoter control. The distribution of genes and repetitive elements within this region, as well as the position of LNCR rasiRNAs expressed during different developmental stages are presented in **Figure 5A**. The inserted TE-LNCR copies are in genomic areas enriched in other repetitive elements (see peaks of repeated elements in **Figure 5A**). The homologous genomic sequences of LNCR rasiRNAs from cluster 1 are present at two distinct locations, whereas homologous genomic sequences of LNCR rasiRNAs from cluster 2 are present at three distinct locations (see gray and white arrows at **Figure 5A**). The precise positions of LNCR rasiRNAs on BAC 83A24 are indicated in **Table S4**.

To monitor the chromatin dynamics that occurs in this genomic region during development, ChIP-qPCR analysis was performed. Twelve primer pairs were designed along the genomic region cloned in BAC 83A24 (see Material and Methods and **Figure 5A**). Primers near the ribosomal gene L37, a highly expressed housekeeping gene, were used as positive controls for the euchromatin marker H3K4me2. Primers at rDNA regions were used as positive controls for heterochromatin H3K9me2 modification [27]. Quantification of immunoprecipitated DNA was performed as described in Material and Methods, and the results of ChIP-qPCR analysis are presented in **Figure 5B**. Interestingly, this region showed a dynamic epigenetic landscape. The heterochromatic marker H3K9me2 is almost absent at this genomic region during early stages of egg development. In contrast, at L2 larval stage, the H3K9me2 marker is abundant and is distributed almost uniformly all over the analysed genomic region. A considerable loss of this heterochromatin marker was detected at the pupal stage, whereas in adults, heterochromatin organization of this genomic region is established again. This analysis revealed the dynamic aspect of heterochromatin marker accumulation related to the development of *S. frugiperda*.

The presence of the euchromatic marker H3K4me2 is also shown, indicating that there is little variation of this euchromatic marker in the analysed genomic region during development. The overall quantity of histone H3 is presented as a control, representing overall variation of histone H3 in the chromatin establishment during different developmental stages. The presence of both euchromatic and heterochromatic markers was detected in a number of loci, which may be caused by the fact that chromatin was prepared from a mixture of different cell types coming from whole insect bodies. Apparently, the rDNA locus in eggs does not contain H3K9me2 heterochromatic marker but there is another locus at the position of primer 7 with statistically significant enrichment of the H3K9me2 marker, confirming that the ChIP analysis was technically appropriate.

### Chromatin organization at the genomic TE-LNCR copies

**Figure 6:**
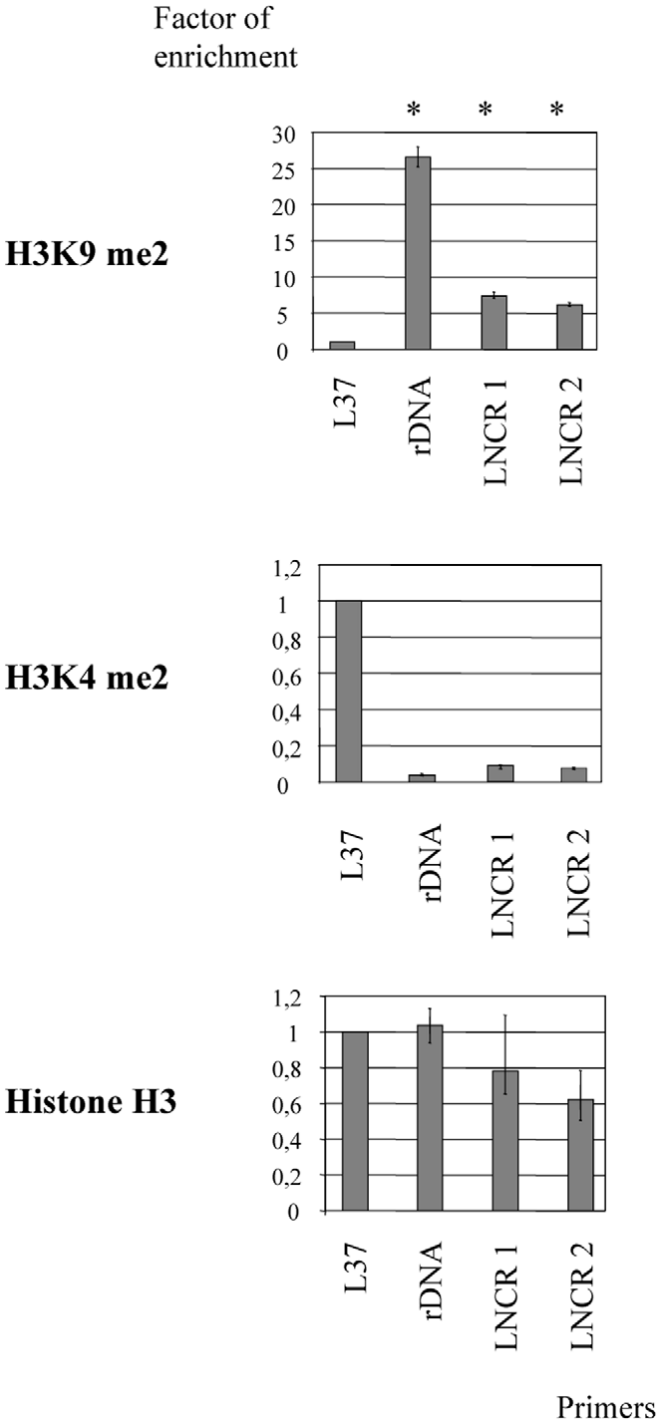
Chromatin organization at the genomic TE-LNCR copies. Presence of histone H3 and histone H3 modifications (H3K9me2 and H3K4me2) at TE-LNCR genomic copies done by ChIP-qPCR. The L37 ribosomal gene was used as a positive control for euchromatic regions and rDNA as a positive control of heterochromatic regions. The chromatin organization at TE-LNCR genomic copies was followed with two pairs of primers (LNCR 1 and 2). Error bars indicate standard deviations over three independent experiments. Asterisks indicate statistically significant enrichment of heterochromatic marker over the control region L37 (p<0.05). P-values were calculated using the Student's t-test.

We next investigated whether the increased H3K9me2 is indeed occurring on the all genomic copies of TE-LNCR at the same time as it was detected in the analysed genomic region of BAC 83A24. To do this, we inspected the epigenetic profile of TE-LNCR copies at the adult developmental stage where formation of heterochromatin was already detected (**Figure 5B**). To evaluate the organisation of chromatin at the TE-LNCR genomic copies, two pairs of primers were designed within the LNCR sequence (LNCR 1 and LNCR 2, see Material and Methods). Chromatin immunoprecipitation (ChIP) was performed with the above mentioned antibodies for modifications of histone H3. The quantity of immunoprecipitated DNA in each sample was evaluated by qPCR and relative enrichment factor was calculated as described in Materials and Methods. Ribosomal gene L37 was used as a positive control for euchromatin and rDNA was a positive control for heterochromatin marker. The calculated relative enrichment factors for two pairs of LNCR primers over the ribosomal L37 genomic locus are presented in **Figure 6**. This analysis shows that H3K9me2 is evenly associated with the TE-LNCR copies in a genome-wide fashion. This indicates that deposition of H3K9me2 on TE-LNCR genomic copies is tightly associated with the act of silencing of this transposable element and formation of the epigenetic landscape. At the same time slight increases of H3K4me2 at TE-LNCR genomic copies (two times enrichment of H3K4me2 over the rDNA loci) could explain the increased level of LNCR expression observed in adults. In summary, it seems that most TE-LNCR genomic copies in adults are in the silenced chromatic state, whereas some of them emerge at active sites, concomitant with increased LNCR expression in adults.

## Discussion

This study shows that post-translational modifications of histones correlate with the expression of a long non-coding (LNCR) and a novel subset of small RNAs (LNCR associated rasiRNAs) in one holocentric species. For the first time, we identified a developmentally regulated heterochromatic region, located at the gene-poor and repeat-rich area of one holocentric species. Accumulation of the heterochromatic histone mark seems to correlate with the appearance of LNCR rasiRNAs, homologous to a long transcript (LNCR) corresponding to an unclassified transposable element. The formation of heterochromatin, the presence of LNCR rasiRNAs and long non-coding RNA (LNCR) are all developmentally regulated. We propose that these heterochromatic areas are involved in transcriptional silencing of TE and discuss that they may also be involved in the developmental regulation of neighbouring genes and/or in putative centromeric chromatin nucleation.

In this study, we initially describe two distinct clusters of *S. frugiperda* LNCR rasiRNAs, probably derived from one long non-coding RNA (LNCR), which seems to operate as an intermediate of unclassified transposable element (TE-LNCR). The Northern blot results are indicating that LNCR transcription starts from multiple genomic sites and that it is probably under control of various external promoters, like it is described for FBPA gene. The other explanation for various LNCR transcript sizes could be also processing of longer LNCR transcripts, so the sequence of LNCR obtained with 454/Roche sequencing that is reported here could be just a contig of overlapping transcripts and processed transcripts.

Surprisingly, most *S. frugiperda* LNCR rasiRNAs are 36 nt long, which, to date, is the longest length of rasiRNAs (piRNAs) reported. In the closely related model, *B. mori*, two classes of small ncRNAs have been described; miRNAs (length 18–32 nt) [28] and piRNAs (length 25–32 nt) [23]. Longer small ncRNAs have not been described yet in *B. mori* and this may be due to gel-size fractionation before sequencing. In the study performed in *B. mori*, RNAs which were 15 to 30 nt long were preselected. In contrast, in the present study, RNAs of 15 to 40 nt in length were preselected before sequencing. Somatic rasiRNAs have not been reported in *B. mori*, but piRNAs isolated from ovary-derived cell lines show unimodal length distribution with a length peak at 27 nt and a strong bias for U at the 5′ end [23]. As reported here, the *S. frugiperda* LNCR rasiRNAs do not have a prominent nucleotide motif. This is in contrast to some previously described rasiRNAs that interact with the Piwi protein [12], [23]. The unusual features of *S. frugiperda* LNCR rasiRNAs suggest a somatic origin and Piwi-protein independent biogenesis for these rasiRNAs. Somatic rasiRNAs produced in a PIWI/AGO3 independent manner have already been demonstrated in *D. melanogaster* COM locus and they operate as silencers of retrotransposons [11]. Because of their size and single-stranded nature, *S. frugiperda* LNCR rasiRNAs also differ from somatic endo siRNAs whose biogenesis depends on AGO2 ([15] for review).

Uni-strand rasiRNAs have been reported to arise mainly from the antisense strand of retrotransposons [4], [29]. Therefore, the described LNCR sequence could be the antisense strand of an atypical, probably non-autonomous retrotransposon. *S. frugiperda* LNCR rasiRNAs are apparently generated from one strand (uni-strand clusters) of LNCR and their biogenesis cannot be assigned to “ping pong” pairing. We presume that some unknown endonuclease activity is involved in the biogenesis of LNCR rasiRNAs (reviewed in [30]). Depending on the developmental stage, different lengths of *S. frugiperda* LNCR rasiRNAs (36 and 33 nt) were detected. We therefore suppose that the endonucleases involved in their biogenesis could be different and specific to certain developmental stages. At the same time, different secondary structures of two LNCR rasiRNA clusters could indicate different functions.

The LNCR rasiRNAs described in this study resemble those described at *Drosophila*'s pericentromeric *flamenco*/COM locus. This locus encodes uni-strand rasiRNAs, corresponding to retrotransposon copies dispersed throughout the *Drosophila* genome and serving as guides, leading to cleavage of expressed retrotransposons in germinal and somatic cells [8], [9]. Families of retroelements distinct from both LTR retrotransposons and non-LTR retrotransposons have been described in many species, and their mechanism of integration is mysterious (reviewed in [31]). The presence of multiple TE-LNCR copies in the *S. frugiperda* genome means that it has, or used to have the ability to transpose. The absence of homology to transposases or reverse transcriptases in the TE LNCR element and the presence of LNCR transcripts, as putative transposition intermediates, suggest that it belongs to class I of TE [32] and that LNCR may use an unknown reverse transcriptase (RT) derived in trans from another genomic source. LNCR RNA could be a retrosequence; a mobile element generated by reverse transcription of mRNA transcripts and subsequent incorporation of the resulting complementary DNA (cDNA) into chromosomal DNA. Retrosequences (also named retroposons), are defined by their inability to encode functional proteins and they have a substantial influence on gene expression patterns, generation of novel gene functions, and genome organization (reviewed in [33]).

Expression of LNCR and diversity of LNCR rasiRNAs are developmentally regulated. A peak of LNCR expression at the L1 larval stage is followed by the generation of a variety of LNCR rasiRNAs in the next larval stage (L2). In the genomic region containing three distinct insertions of TE-LNCR, an increase of the heterochromatic marker H3K9me2 is observed at the L2 larval stage compared with the previous developmental stage (eggs). Another peak of LNCR expression occurs at the pupal stage, but the quantity and diversity of LNCR rasiRNAs is low and the loss of heterochromatic markers can be observed at the analyzed genomic region. Finally, in adults, increased expression of LNCR and heterochromatin formation in analysed genomic regions is again evident. These dynamic chromatin states and LNCR expression suggest that LNCR accumulates at the L1 stage in a sufficient amount to generate LNCR rasiRNAs and heterochromatin at the later L2 stage. In the pupal stage there is an increased expression of LNCR, but the amount or diversity of LNCR rasiRNAs may still be insufficient to generate heterochromatin. Finally, in adults LNCR expression is increased and heterochromatin is established again but there is no data concerning adult LNCR rasiRNA expression. As a hypothesis, we propose that accumulation of heterochromatin mark is shortly delayed in respect to increased LNCR expression and generation of LNCR rasiRNAs. However, we have still no evidence to demonstrate a direct link between these three steps.

The TE-LNCR copy, inserted in the fourth intron of the FBPA (fructose 1–6 biphosphate aldolase) gene or other genes, could be co-transcribed with that gene. As a hypothesis, we propose that the resulting transcript may be a substrate for generation of LNCR rasiRNAs which could guide heterochromatin formation locally or at other TE-LNCR locations in the genome. Since the FBPA expression pattern does not correspond completely to the LNCR expression pattern (Solexa high throughput sequencing of RNA expressed in all developmental stages, unpublished data), we assume that this is not the only possible site of LNCR transcription and that there are probably various LNCR transcription sites in the *S. frugiperda* genome.

LNCR rasiRNAs are presumably involved in RNA-guided sequence-specific establishment and/or maintenance of heterochromatin, but they may also contribute to guided RNA degradation. Heterochromatin-mediated silencing has been studied during *D. melanogaster* development in transgenic lines exhibiting position effect variegation (PEV) of the reporter gene. In mitotically active cells of imaginal disks, silencing of the reporter gene increases in the embryo after the 14^th^ cell cycle then reaches a plateau during the three larval stages and decreases again at the pupal stage during which differentiation occurs [34], [35]. The level of heterochromatic marks measured on the 24 follows a similar kinetic during *S. frugiperda* development. In addition to TE silencing, formation of heterochromatin in this region could be involved in the epigenetic control of the genes placed in this area.

At the cytological level, heterochromatin is visible at the tips of autosomes and/or on sexual chromosomes [36]–[38], but not at the centromeric regions which are dispersed along the chromosome length in holocentric insects [21], [39]. However, the heterochromatic landscape remains unclear at the molecular level in holocentric chromosomes. The present study shows the existence of transient heterochromatic regions correlated with the appearance of a long ncRNA (LNCR) and homologous LNCR rasiRNAs. Although centromeres in monocentric chromosomes are usually fixed - except in the case of neocentromere formation - we speculate that these heterochromatic regions could be involved in the organisation of *S. frugiperda* holocentromeres. In the fission yeast *Schizosacharomyces pombe*, long double-stranded RNAs arising from bidirectional transcription of centromeric repeats, give rise to small dsRNAs (22–25 nt long) which target the formation and maintenance of heterochromatin at the centromere [40], [41], [42]. LINE retrotransposon RNA is an essential structural and epigenetic component of a core human neocentromere [43]. In human centromeres, satellite RNA is a key component for the assembly of kinetochore and RNase treatment delocalizes CENP-C from mature kinetochore [44]. Recently, Bergman *et al.* showed that inhibition of alpha satellites transcription by H3K4 demethylation prevents loading of CENP-A at a human centromere by CENP-A chaperone HJURP [45]. Single-stranded nucleic acids (but no siRNAs) of 40 to 200 nt in length have been shown to promote maize CENP-C DNA binding activity and these centromeric rasiRNAs remain tightly bound to maize centromeric chromatin [46], [47]. In rice, siRNAs of 21–24 nt in length and homologous to the CentO centromeric repeat have been detected by Northern blots with reverse and forward centromeric repeats as probes [48]. In the same study, a small RNA band of 40 nt was detected, matching mostly with the reverse probe and corresponding probably to single-stranded RNA molecules. Recently, rasiRNA of 34–42 nucleotides derived from transcription of a satellite and a retroviral sequence located in the centromere of a mammalian species have been described [49] and are termed “crasiRNA” (centromere repeat-associated small interacting RNA). The LNCR rasiRNA described in our paper may belong to this new class. In *S. frugiperda*, centromere location may fluctuate according to developmental programming and rasiRNAs could guide their formation. The absence of homologs for the major centromeric histone H3-like proteins CENP-A and CENP-C in *S. frugiperda* transcriptome sequences (unpublished data), as well as in the close lepidopteran model genome *B. mori* [50] makes it difficult to evaluate a link between these heterochromatin domains and rasiRNAs with holocentric centromere. Nevertheless, the putative role of LNCR rasiRNAs in centromere formation would be a matter of future investigation.

## Materials and Methods

### RNA isolation and preparation of small RNA libraries for deep sequencing

Total RNA was isolated from three developmental stages of *Spodoptera frugiperda* (2.5 days old fertilized eggs, L2 larval stage and 12 days old pupae) using mirVana isolation kit (Ambion) according to manufacturer instructions. We have started from 2000–3000 eggs, 300 L2 larvae and 6 pupae – 3 females and 3 males. 20 micrograms of total RNA was separated on denaturing polyacrylamide gel and small RNAs ranging from 15 to 40 nt were extracted. RNA adaptor (5′ GUUCAGAGUUCUACAGUCCGACGAUC) was ligated at 5′end of small RNAs with RNA ligase and than size selected (40 and 60 nt) after electrophoresis on 15% denaturing polyacrylamide gel (TBE). After that 3′ adaptor (5r**A**pp/ATCTCGTATGCCGTCTTCTGCTTG/3dd**C**/) was ligated. 3′ adaptor has adenylated RNA base at the 5′ end and a dideoxy C base at the 3′ end to block mis-ligation to the 5′ end of small RNAs. After ligation of adaptors on both ends, RNA size between 70 and 90 nt was isolated from 10% denaturing polyacrylamide gel.

Reverse transcription was done with primer 5′AGCATACGGCAGAAGACGAAC and subsequent PCR with primers 5′AGCATACGGCAGAAGACGAAC and 5′ AATGATACGGCGACCACCGACAGGTTCAGAGTTCTACAGTCCGA. This protocol selectively enriches those RNA fragments that have adaptor molecules on both ends. cDNA constructs were purified after 12 cycles of PCR amplification on 6% polyacrylamide gel (TBE) and sequenced with primer 5′ CGACAGGTTCAGAGTTCTACAGTCCGACGATC using SOLEXA sequencing technology (Illumina Inc. Hayward, CA). The preparation of small RNA libraries and Illumina sequencing was done once.

### Computational analysis of small RNA sequencing data

Small RNA reads, up to 44 nt in length, were produced using a Genome Analyzer 1G (Illumina Inc., Hayward, CA). Low quality reads were trimmed and excluded. The 3′adaptor sequences (exact or degenerated) were identified and accurately clipped with the aid of a house made dynamic programming algorithm (Skuldtech). Reads without adaptor sequences were discarded. All the homopolimers (polyA, polyU, polyC and polyG) longer than 12 nt were trimmed and only the sequences longer than 10 nt were kept. After elimination of redundancy, sequences were mapped to the available genomic sequences cloned in 37 BACs (1% of *S. frugiperda's* genome), RNA libraries and the annotated DNA repeated elements using BLASTn, allowing no mismatch (100% of sequence identity all along their length). The sequences of small RNAs with 100% of sequence identity all along their length were used for further analysis. Small RNA sequences matching perfectly to *S. frugiperda* rDNA and sequences with more than 95% similarity with *Drosophila melanogaster* tRNA (*S. frugiperda* tRNA are not yet identified) were used as control of RNA degradation.

The p-value for each small RNA copy number was calculated (each library was compared with two others). However, all LNCR rasiRNA sequences that have been acquired are presented regardless of their p-value, in order to preserve qualitative data (for example, LNCR rasiRNA Sf_0483512 is found in only one copy in pupal stage and no copies in other two libraries, meaning that its p>0.05).

Sequences of LNCR rasiRNAs from different samples (libraries) were normalized by the number of total reads and presented as percentage of total high quality reads in each library. For example, sequence of LNCR rasiRNA with ID Sf_0291303 was found in 167 copies in eggs library, which makes 3.089 10^−3^% of total high quality sequences found in eggs (5,405,284). At the same time this sequence was found in 5 copies in L2 small RNA library, making 0.088 10^−3^% of the total of 5,678,498 high quality RNA sequences detected in this developmental stage etc. Since endogenous small RNA control, that is equally expressed in all three developmental stages, is not known, this was the only way to normalise expression of small RNA in three libraries. Graph in Figure 2C was made as sum of all LNCR rasiRNA copies found in each developmental stage (total of 1921, 12238 and 120 LNCR rasiRNA copies in eggs, L2 and pupal library, respectively) and presented as percentage over the total number of high quality sequences in each developmental stage (5405284, 5678498 and 11167 633 respectively).

### Northern blot

15 micrograms of total RNA, extracted from the *S. frugiperda* adults, was mixed with equal volume of loading buffer (95% formamide, 18 mM EDTA, and 0.025% SDS, Xylene Cyanol, and Bromophenol Blue). The sample was denatured at 95°C for 5 min and separated on 1.2% agarose gel, together with RNA ladder (Transcript RNA Marker, 0.2–10 kb Sigma Aldrich). After electrophoresis the gel was stained with EtBr, distained and the separated RNAs are transferred to a Nylon SPC membrane (Whatman) by capillary transfer in 20×SSC. After transfer the RNA was fixed by exposure to UV light.

The probe of 1244 bp was generated by PCR, using 250 ng of *S. frugiperda* adult cDNA as a template and primers ncRNA_long. This DNA probe was labelled by random primed labelling in the presence of ^32^P dCTP, purified with Illustra Microspin G25 column (GE Healthcare) from free nucleotides and denatured before hybridisation. Hybridisation was done over night at 42°C in hybridisation buffer (5×SSC, 5× Denhardt, 50% formamide, 1% SDS). The membrane was washed after hybridisation as follows: two times for 15 min at 42°C with 2×SSC/0.1% SDS, two times for 15 minutes at 42°C with 0.2×SSC/0.1% SDS and once in 0.2×SSC at room temperature.

### Nucleotide sequence accession number

The nucleotide sequences reported in this study have been submitted to the DDBJ/EMBL/GenBank data bank under accession number listed in the Table S2.

### Chromatin immunoprecipitation (ChIP)

Chromatin was prepared from. 2.5-day old fertilized eggs, L2 larval stage, and 12-day old pupae of *Spodoptera frugiperda* as previously described (http://www.igh.cnrs.fr/equip/cavalli/Lab%20Protocols/Cavalli_lab_ChIP.pdf). We started from 0.2 g of embryos or L2 larvae, or 0.6 g of pupae (one female and one male). The starting biological material was frozen in liquid nitrogen, and was then homogenized in buffer A1 (60 mM KCl, 15 mM NaCl, 4 mM MgCl2, 15 mM HEPES (pH 7.6), 0.5% Triton X-100, 0.5 mM DTT, EDTA-free protease inhibitor cocktail (Roche)) with 1.8% formaldehyde for 15 min at RT. Cross linking was stopped by addition of glycine to give a final concentration of 0.25 M (5 min on ice). Nuclei were precipitated by centrifugation (5 min, 4000 g at 4°C), washed 3 times with buffer A1 and once with Lysis Buffer without SDS (140 mM NaCl, 15 mM HEPES [pH 7.6], 1 mM EDTA [pH 8], 0.5 mM EGTA, 1% Triton X-100, 0.5 mM DTT, 0.1% Sodium deoxycholate, EDTA-free protease inhibitor cocktail [Roche]). Then we resuspended pellets in 1.5 ml Lysis Buffer with 0.1% SDS, 0.5% N-Laurosylsarcosine and incubated them for 30 min at 4°C on a rotating wheel. Chromatin was sheared on ice by sonication to DNA fragments from 200 bp to 800 bp and then centrifugated 3 times for 5 min at 14 000 rpm at 16°C to remove insoluble material from chromatin. Preclearing of the chromatin with A/G sepharose beads was done on a rotating wheel overnight at 4°C. We used chromatin from 5 to 20 mg of starting biological material for one ChIP reaction.

For immunoprecipitation of chromatin we use rabbit polyclonal anti histone H3 (dimethyl Lys4) antibody (Upstate 07-030), rabbit monoclonal anti dimethyl histione H3 (Lys4) antibody (Abcam32356, Y47), mouse monoclonal anti histone H3 (di methyl K9) antibody (Abcam 1220) and rabbit polyclonal anti histone H3 antibody (Abcam1791). The optimal concentration of antibodies was determined experimentally and then incubation of chromatin and antibodies was done for 8 h on rotating wheel at 4°C. Control sample (mock) was without antibodies. Then we added protein A/G beads and incubation was prolonged over night.

The samples were washed 4× with 1 ml Lysis buffer (0.05% SDS+PI) and 2× with 1 ml TE buffer. Each wash is for 5 to 10 min at 4°C on a rotating wheel and centrifugation is for 1 min at 400 g at 4°C. The chromatin was eluted from beads by adding 250 µl Elution buffer (10 mM EDTA, 1% SDS, 50 mM Tris-Cl pH 8) and incubation for 30 min at 65°C with vigorous shaking. Decrosslinking was done by incubation of the samples ON at 65°C. After decrosslinking samples were treated with proteinase K for 3 h at 50°C, LiCl (0.7 M final concentration) was added and DNA fragments were purified with phenol/chlorophorm/isoamylalcohol mixture. DNA fragments were precipitated with cold ethanol and resuspended in 200 µl of TE buffer.

### Quantitative PCR

The quantity of immunoprecipitated DNA in each ChIP experiment was determined first as an absolute amount compared with a standard curve made by serial dilutions of sheared *S. frugiperda* genomic DNA. The standard curve was made separately for each set of primers. After that the quantity of immunoprecipitated DNA was presented as a percentage of input (starting) DNA. To compare the results from independent ChIP experiments, all the data are presented as a factor of enrichment relative to the amount of DNA that was measured with primer set Sf_L37 (placed at the proximity of ribosomal gene L37) and taken as 1.

Quantitative PCR was done with the following pairs of primers:
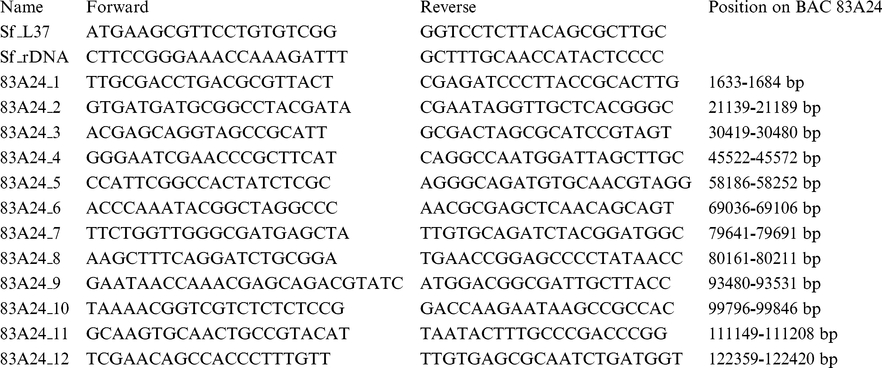


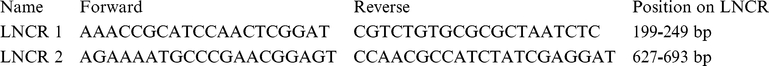



### RT PCR

Total RNA was isolated from different developmental stages (2.5 days old eggs, L1–L6 larval stages, 12 days old pupae and adults) of *S. frugiperda*. cDNA preparation was done with SuperScript® III Reverse Transcriptase (Invitrogen) kit according to the manufacturer's instructions. Semi quantitative PCR was done with the following pairs of primers:
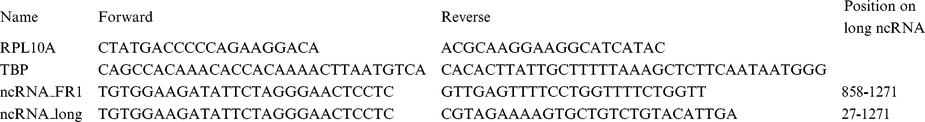



### Western blotting

Nuclear protein extracts prepared from different developmental stages of *S. frugiperda* were separated by 15% SDS/PAGE. Primary antibodies used for the detection were rabbit polyclonal anti histone H3 (dimethyl Lys4) antibody (Upstate 07-030), rabbit monoclonal anti dimethyl histione H3 (Lys4) antibody (Abcam32356, Y47), mouse monoclonal anti histone H3 (di methyl K9) antibody (Abcam 1220) and rabbit polyclonal anti histone H3 antibody (Abcam1791).

The quantification of Western-blot signals was done with ImageQuant TL software on three independent Western-blots exposures obtained in the linear range. The signal intensity of each band was determined using volume analysis with object average background correction applied. Data were exported to Microsoft Excel. The histone H3 was taken as the reference protein. The volume signal of histone H3 present in E1 eggs (1 day old fertilized eggs) was taken as 1 and the other H3 signal volumes were calculated relative to this. The volume signals of H3K9 and H3K4 were normalized after that, relative to the presence of histone H3 in each developmental stage. The normalized H3K9 and H3K4 signal volumes in E1 eggs (1 day old fertilized eggs) were taken as 1 and the rest of the signal volumes were calculated relative to this.

### Silver staining of polyacrylamide minigels

The total RNA was run on 17% polyacrylamide gels containing 7 M urea. After the electrophoresis run, the gel was rinsed in bidistilled water, RNA was fixed in 10% ethanol for 15 minutes and the gel was rinsed again in bidistilled water. The gel was incubated in 1% nitric acid for 5 minutes, rinsed three times in bidistilled water and incubated in 0.2% silver nitrate for 30 minutes. The gel was rinsed extensively in bidistilled water and then developed in 3% sodium carbonate/0.05% formaldehyde, until the desired intensity of bands and background was reached. The reaction was stopped in 3% glacial acetic acid.

## Supporting Information

Figure S1 **The quality of total and small RNA isolated at different developmental stages of** ***S. Frugiperda*** **.** Total RNA isolated from whole *S. frugiperda* body at three developmental stages (2.5-day old fertilised eggs, L2 larval stage and 12 days old pupae) was separated on A) 1% native agarose gel and stained with EtBr; B) 17% polyacrylamide/7 M urea denaturing gel and stained with silver.
(TIF)

Figure S2 **Alignment of** ***S. frugiperda*** **LNCR rasiRNAs with long non coding RNA (LNCR).** The sequence of LNCR is on the lower panel. The positions of cluster 1 and 2 LNCR rasiRNAs are labelled in red.
(TIF)

Figure S3 **Predicted secondary structures of** ***S. frugiperda*** **LNCR rasiRNAs.** The LNCR rasiRNA sequences are folded on Mfold web server for nucleic acid folding and hybridization prediction - M. Zuker (http://mfold.bioinfo.rpi.edu/cgi-bin/rna-form1.cgi), using the default settings. Predicted secondary structure with the lowest free energy is presented. Free energy values vary from dG = −1.00 to dG = −5.60 kcal/mol.
(TIF)

Figure S4 **Expression analysis of** ***S. frugiperda*** **long ncRNA (LNCR) during different developmental stages done by qRT PCR.** Graphs present the relative LNCR copy number during development relative to the expression of ribosomal gene RPL37 used as endogenous control gene (E2- 2.5-day old fertilized eggs, L1–L6 larval stages, P- 12 days old pupae and A- adults). qRT PCR was performed on RNA samples treated with (RT+) or without reverse transcriptase (RT−). qRT PCR was done with the LNCR 1, LNCR 2 and Sf_L37 pair of primers. All runs were performed using Roche LC 480 detection system in a 10 µl reaction containing 50 ng of cDNA. The qRT-PCR reaction conditions were as follows: 95°C for 5 min, followed by 40 cycles of 95°C for 10 s, 60°C for 10 s and 72°C for 10 s. Transcripts were quantified as follows: the threshold cycle (Ct) is defined as the cycle number at which the quantity of fluorescence product passes a pre-determined threshold. The relative amounts were calculated using the equation: ΔCt = Ct RPL37−Ct LNCR 1 or ΔCt = Ct RPL37−Ct LNCR 2. ΔCts were then converted to relative copy numbers with the formula 2^ΔCt^.
(TIF)

Figure S5 **The presence of histone H3 modification during different developmental stages of** ***S. frugiperda*** **.** A) Western blot showing the presence of histone H3 modification during the different developmental stages of *S. frugiperda* (E1- 1 day old eggs, E2- 2.5 days old eggs, L1–L6 larval stages, P- 12 days old pupae and A-adults). B) Graph presenting the normalized quantity of H3K4me2 and H3K9me2 Western-blot signals relative to the total quantity of histone H3. Quantification was performed with ImageQuant TL software on three independent Western blots. Error bars indicate standard deviations. P-values were calculated using the Student's paired t-test.
(TIF)

Table S1 The flow results of data filtration and distribution of sequenced small RNAs in three developmental stages of *S. frugiperda* (2.5 days old fertilized eggs, L2 larval stage and 12 days old pupae). The raw data in each library presents the sum of low and high quality reads. Low-quality reads were excluded from further analysis. High-quality reads present the sequences longer than 10 nt, without homopolymers (>12 nt), and with clearly detected and clipped adaptor sequences. Unique sequences present the number of diverse sequences in each library.
(PPTX)

Table S2 Accession numbers of 46 LNCR rasiRNAs, 11 TE LNCR copies and LNCR.
(PPTX)

Table S3 Table of *S. frugiperda* LNCR rasiRNAs. LNCR rasiRNAs are sorted in cluster 1 and cluster 2 with their names, sequences, percentage in each of three libraries (2.5 days old fertilized eggs, L2 larval stage and 12 days old pupae) and their position on LNCR and the consensus DNA repeated element (TE LNCR consensus or Spodo2-B-R19-Map11_NoCat-consensus (LepidoDB)).
(PPTX)

Table S4 Mapping of LNCR rasiRNAs to genomic regions. Position of the LNCR rasiRNAs on some of the *S. frugiperda* genomic regions that are cloned in bacterial artificial chromosome (BAC) and sequenced. Only perfect matches are presented.
(PPTX)
