# Supplementary figures and images for: Correlation of LNCR rasiRNAs Expression with Heterochromatin Formation during Development of the Holocentric Insect Spodoptera frugiperda
Source: PLoS One. 2011 Sep 30;6(9):e24746. doi: 10.1371/journal.pone.0024746 (PMC3184123; doi:10.1371/journal.pone.0024746)

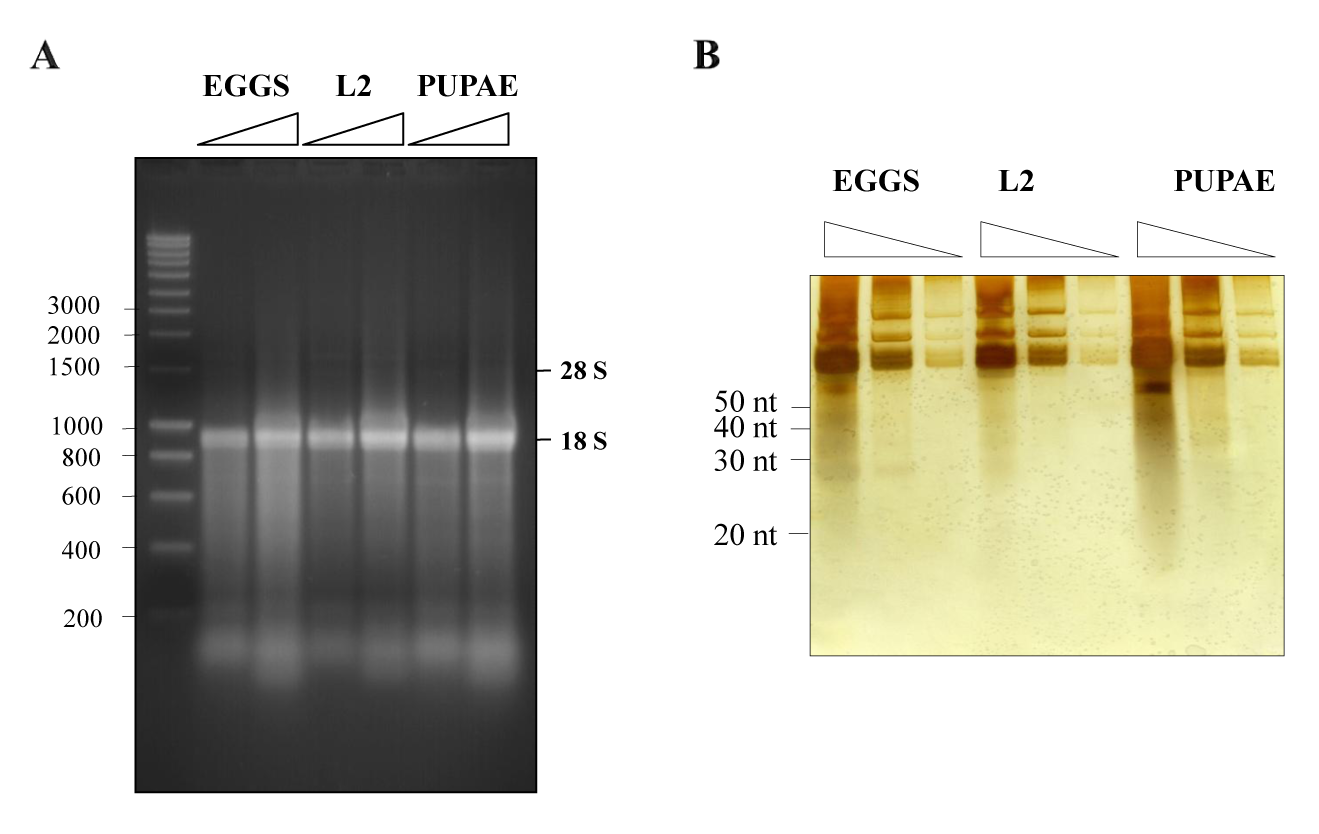

Supplement: Figure S1 — The quality of total and small RNA isolated at different developmental stages of S. Frugiperda . Total RNA isolated from whole S. frugiperda body at three developmental stages (2.5-day old fertilised eggs, L2 larval stage and 12 days old pupae) was separated on A) 1% native agarose gel and stained with EtBr; B) 17% polyacrylamide/7 M urea denaturing gel and stained with silver. (TIF) [file pone.0024746.s001.tif]

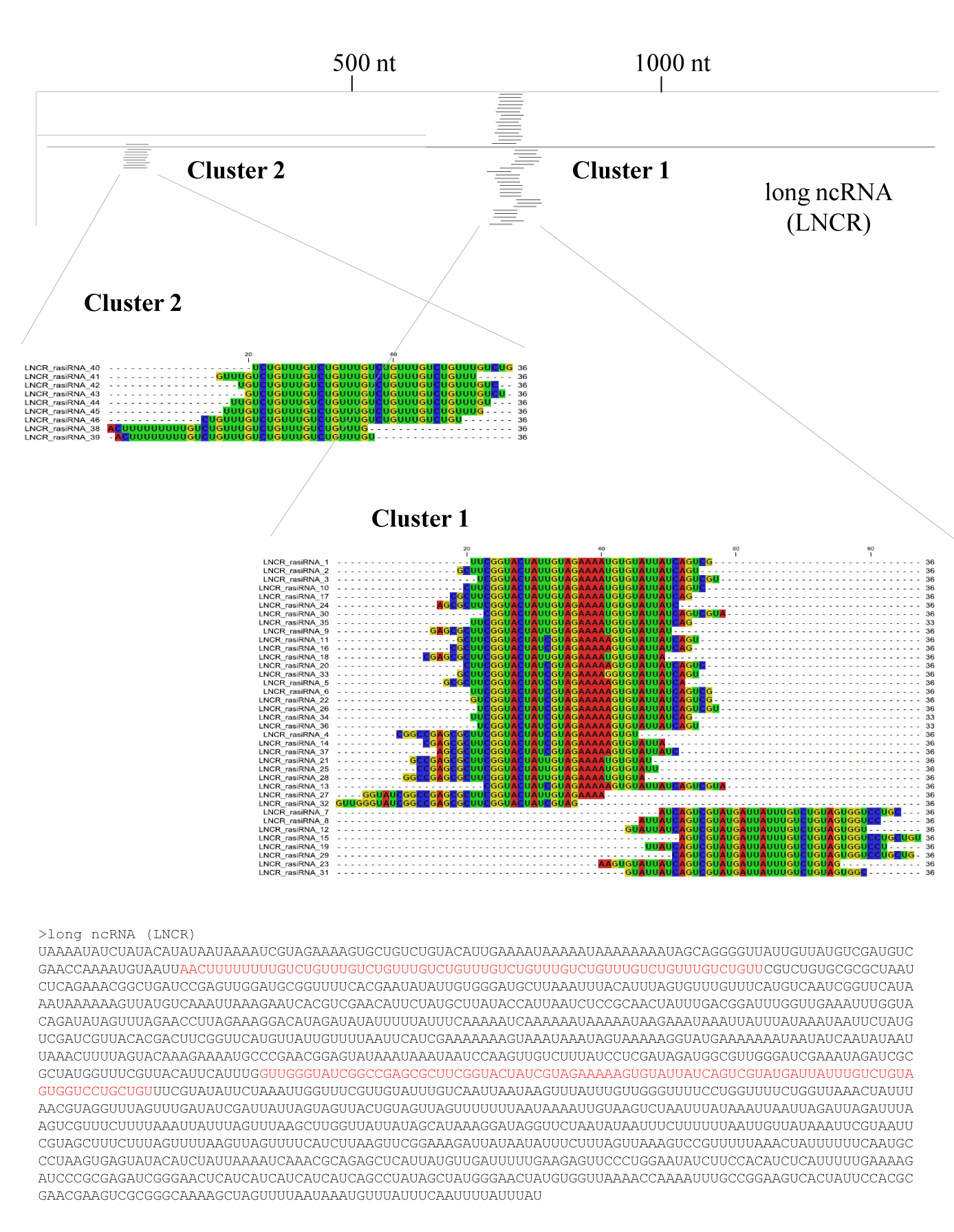

Supplement: Figure S2 — Alignment of S. frugiperda LNCR rasiRNAs with long non coding RNA (LNCR). The sequence of LNCR is on the lower panel. The positions of cluster 1 and 2 LNCR rasiRNAs are labelled in red. (TIF) [file pone.0024746.s002.tif]

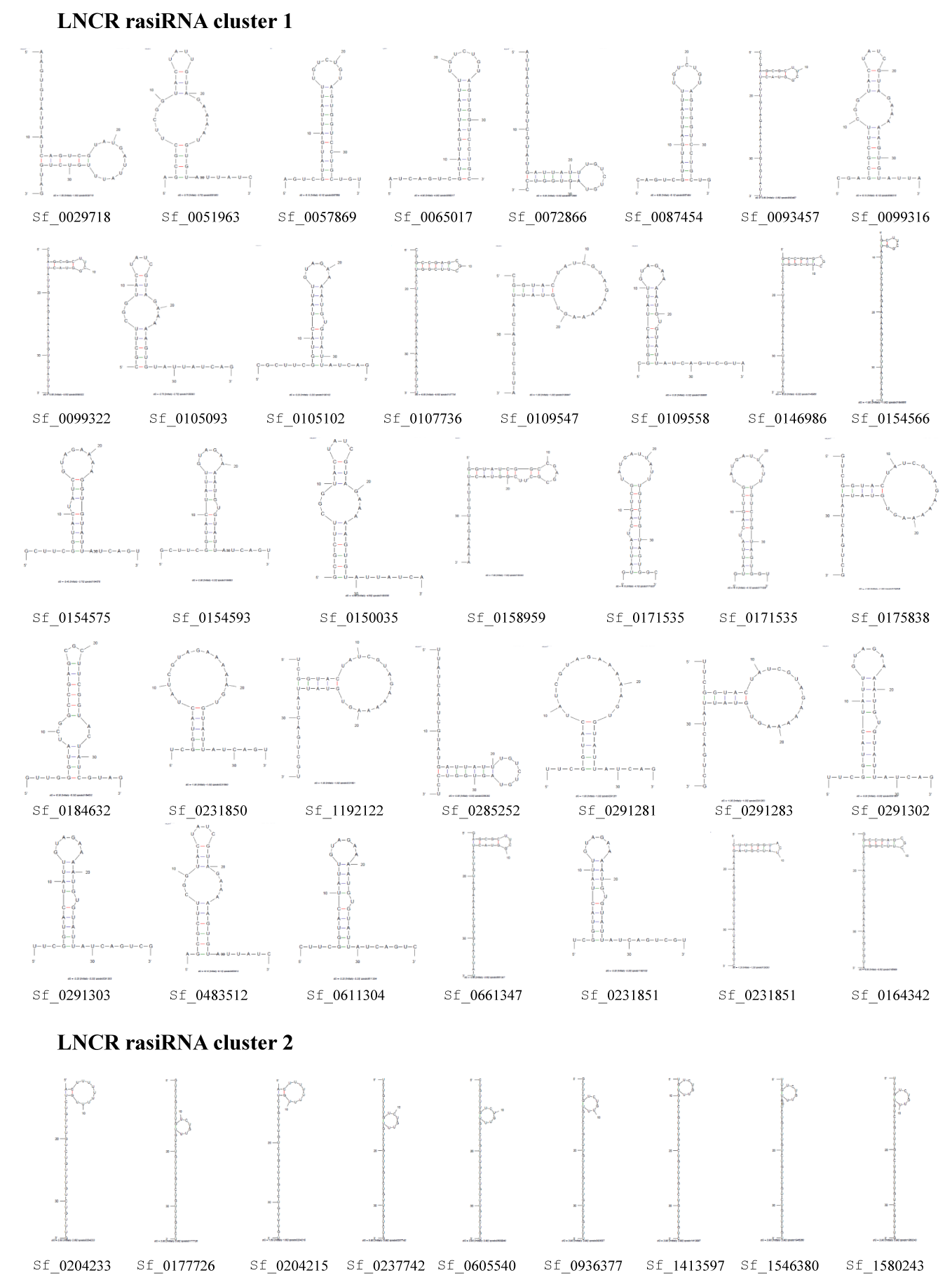

Supplement: Figure S3 — Predicted secondary structures of S. frugiperda LNCR rasiRNAs. The LNCR rasiRNA sequences are folded on Mfold web server for nucleic acid folding and hybridization prediction - M. Zuker (http://mfold.bioinfo.rpi.edu/cgi-bin/rna-form1.cgi), using the default settings. Predicted secondary structure with the lowest free energy is presented. Free energy values vary from dG = −1.00 to dG = −5.60 kcal/mol. (TIF) [file pone.0024746.s003.tif]

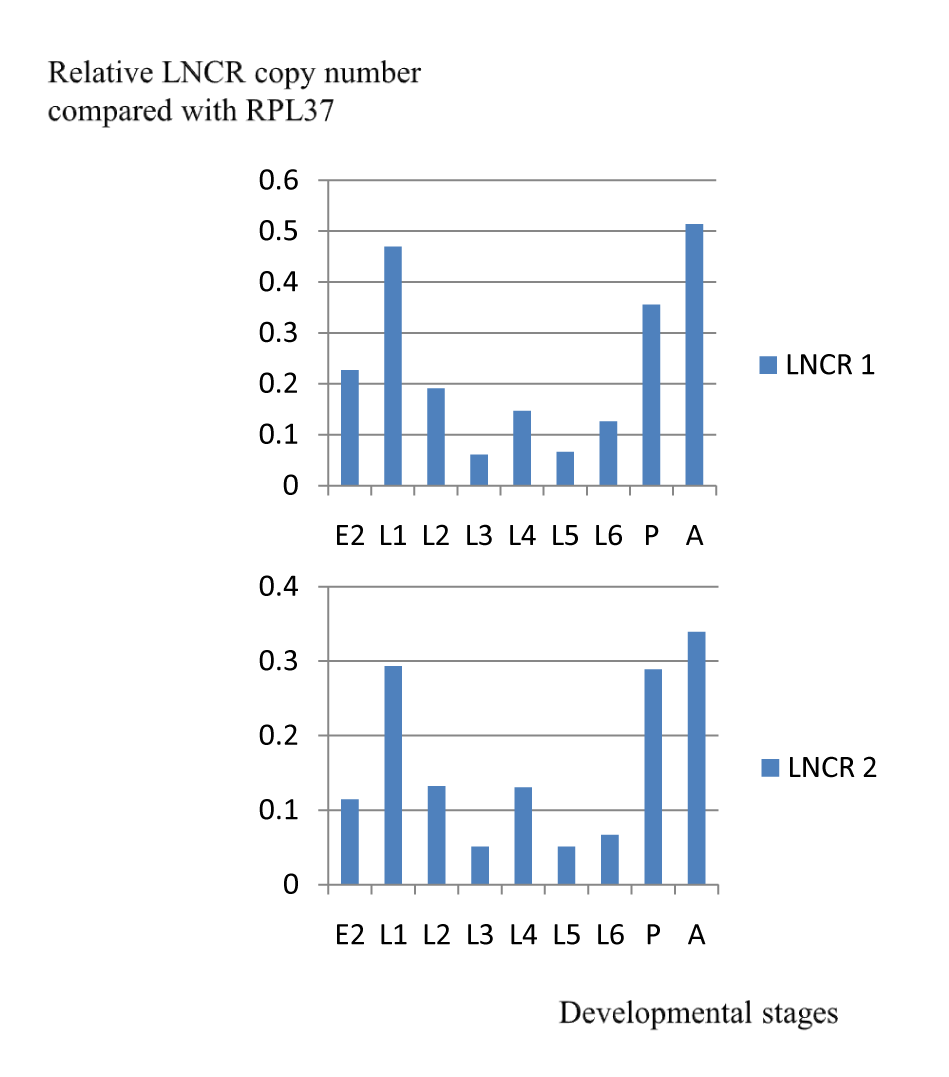

Supplement: Figure S4 — Expression analysis of S. frugiperda long ncRNA (LNCR) during different developmental stages done by qRT PCR. Graphs present the relative LNCR copy number during development relative to the expression of ribosomal gene RPL37 used as endogenous control gene (E2- 2.5-day old fertilized eggs, L1–L6 larval stages, P- 12 days old pupae and A- adults). qRT PCR was performed on RNA samples treated with (RT+) or without reverse transcriptase (RT−). qRT PCR was done with the LNCR 1, LNCR 2 and Sf_L37 pair of primers. All runs were performed using Roche LC 480 detection system in a 10 µl reaction containing 50 ng of cDNA. The qRT-PCR reaction conditions were as follows: 95°C for 5 min, followed by 40 cycles of 95°C for 10 s, 60°C for 10 s and 72°C for 10 s. Transcripts were quantified as follows: the threshold cycle (Ct) is defined as the cycle number at which the quantity of fluorescence product passes a pre-determined threshold. The relative amounts were calculated using the equation: ΔCt = Ct RPL37−Ct LNCR 1 or ΔCt = Ct RPL37−Ct LNCR 2. ΔCts were then converted to relative copy numbers with the formula 2ΔCt. (TIF) [file pone.0024746.s004.tif]

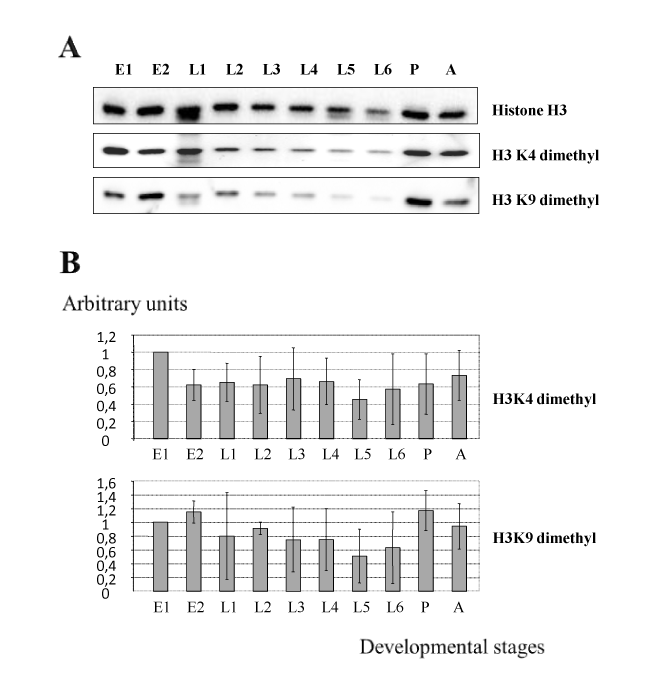

Supplement: Figure S5 — The presence of histone H3 modification during different developmental stages of S. frugiperda . A) Western blot showing the presence of histone H3 modification during the different developmental stages of S. frugiperda (E1- 1 day old eggs, E2- 2.5 days old eggs, L1–L6 larval stages, P- 12 days old pupae and A-adults). B) Graph presenting the normalized quantity of H3K4me2 and H3K9me2 Western-blot signals relative to the total quantity of histone H3. Quantification was performed with ImageQuant TL software on three independent Western blots. Error bars indicate standard deviations. P-values were calculated using the Student's paired t-test. (TIF) [file pone.0024746.s005.tif]
