# Supplementary material for: Correlation of LNCR rasiRNAs Expression with Heterochromatin Formation during Development of the Holocentric Insect Spodoptera frugiperda
Source: PLoS One. 2011 Sep 30;6(9):e24746. doi: 10.1371/journal.pone.0024746 (PMC3184123; doi:10.1371/journal.pone.0024746)
Supplement: Table S1 — The flow results of data filtration and distribution of sequenced small RNAs in three developmental stages of S. frugiperda (2.5 days old fertilized eggs, L2 larval stage and 12 days old pupae). The raw data in each library presents the sum of low and high quality reads. Low-quality reads were excluded from further analysis. High-quality reads present the sequences longer than 10 nt, without homopolymers (>12 nt), and with clearly detected and clipped adaptor sequences. Unique sequences present the number of diverse sequences in each library. (PPTX) [file pone.0024746.s006.pptx]

## Slide 1
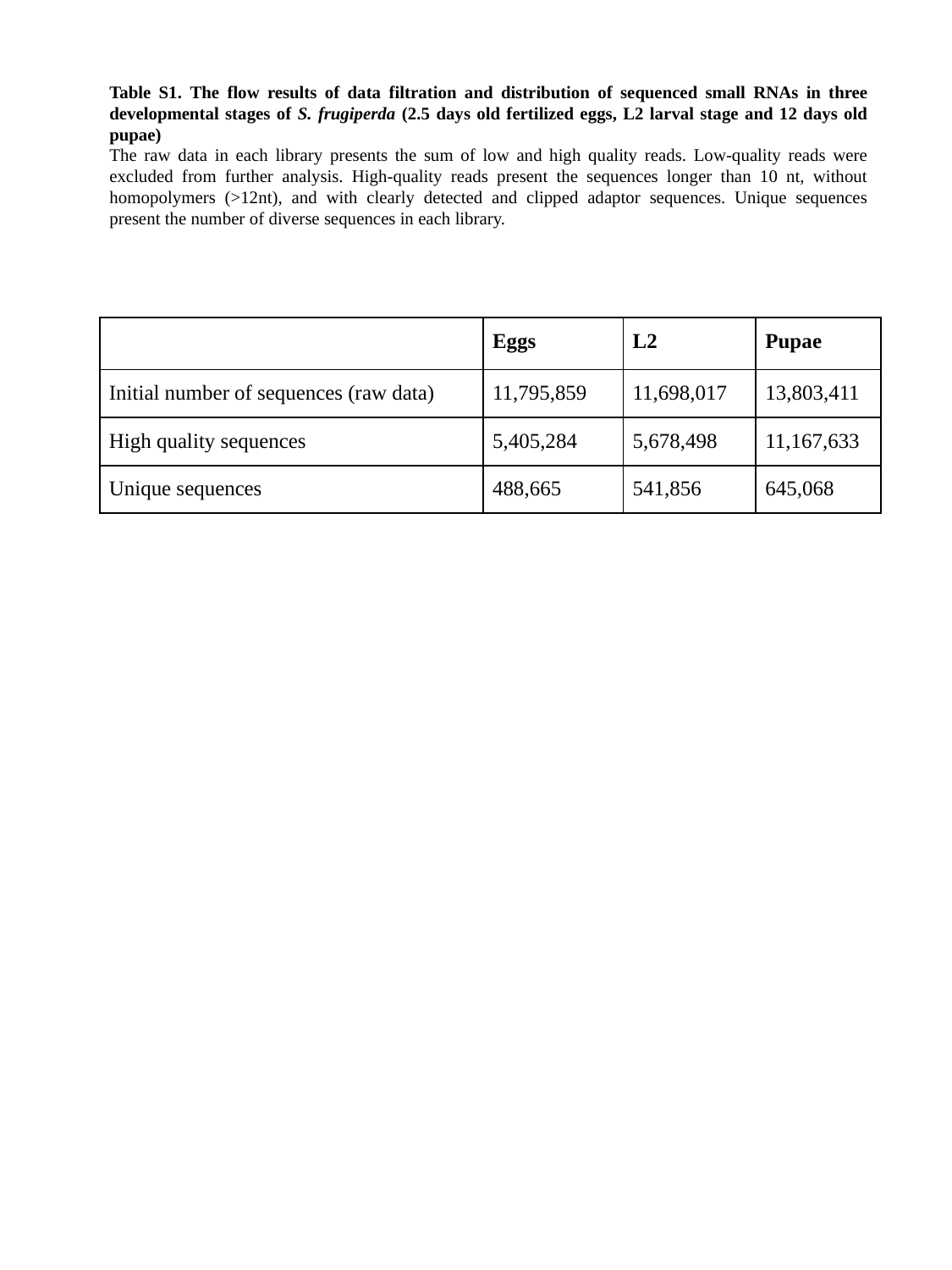

Table S1. The flow results of data filtration and distribution of sequenced small RNAs in three developmental stages of S. frugiperda (2.5 days old fertilized eggs, L2 larval stage and 12 days old pupae)
The raw data in each library presents the sum of low and high quality reads. Low-quality reads were excluded from further analysis. High-quality reads present the sequences longer than 10 nt, without homopolymers (>12nt), and with clearly detected and clipped adaptor sequences. Unique sequences present the number of diverse sequences in each library.
| | Eggs | L2 | Pupae |
| --- | --- | --- | --- |
| Initial number of sequences (raw data) | 11,795,859 | 11,698,017 | 13,803,411 |
| High quality sequences | 5,405,284 | 5,678,498 | 11,167,633 |
| Unique sequences | 488,665 | 541,856 | 645,068 |
