# Supplementary material for: Correlation of LNCR rasiRNAs Expression with Heterochromatin Formation during Development of the Holocentric Insect Spodoptera frugiperda
Source: PLoS One. 2011 Sep 30;6(9):e24746. doi: 10.1371/journal.pone.0024746 (PMC3184123; doi:10.1371/journal.pone.0024746)
Supplement: Table S2 — Accession numbers of 46 LNCR rasiRNAs, 11 TE LNCR copies and LNCR. (PPTX) [file pone.0024746.s007.pptx]

## Slide 1
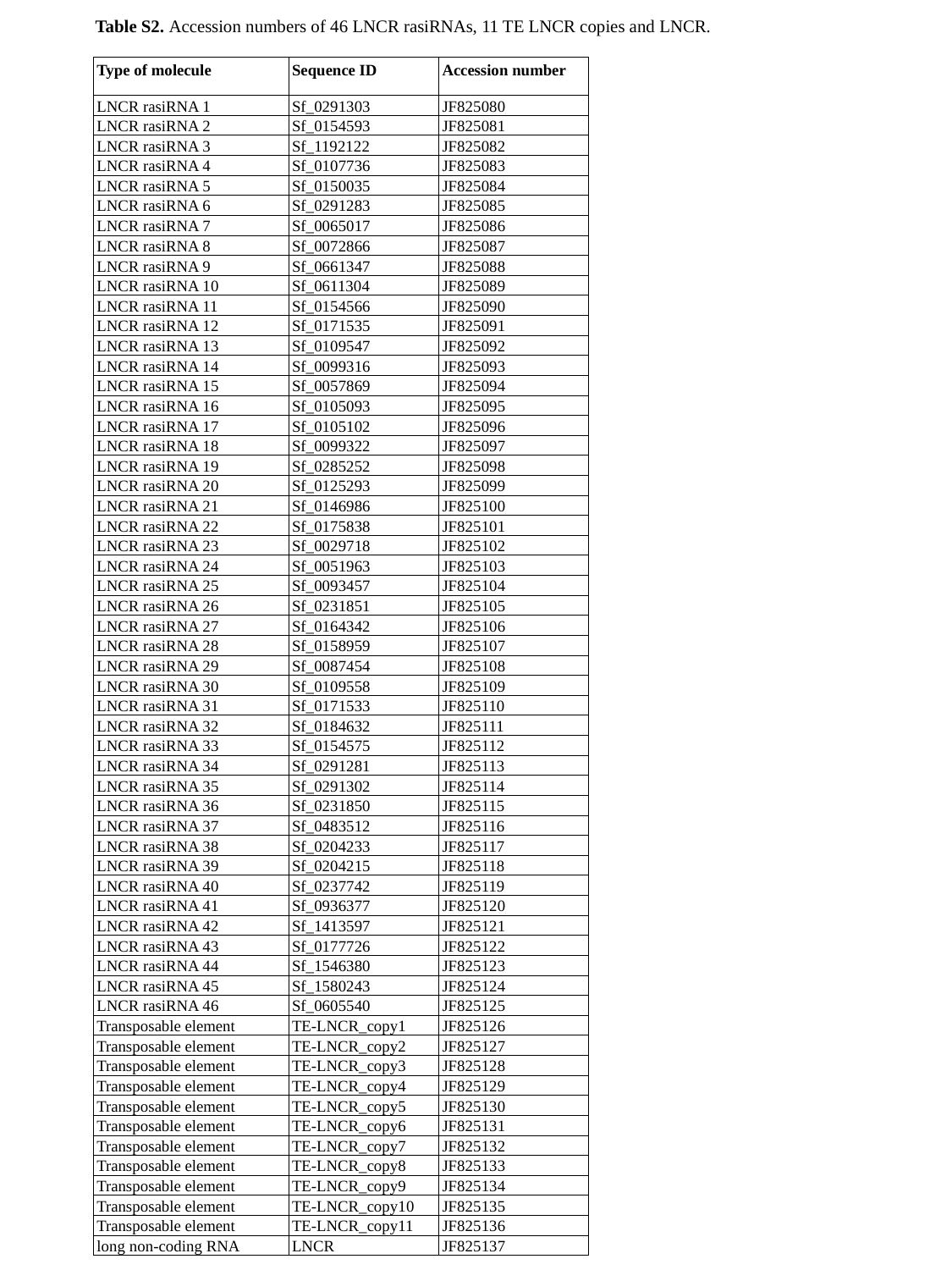

Table S2. Accession numbers of 46 LNCR rasiRNAs, 11 TE LNCR copies and LNCR.
| Type of molecule | Sequence ID | Accession number |
| --- | --- | --- |
| LNCR rasiRNA 1 | Sf\_0291303 | JF825080 |
| LNCR rasiRNA 2 | Sf\_0154593 | JF825081 |
| LNCR rasiRNA 3 | Sf\_1192122 | JF825082 |
| LNCR rasiRNA 4 | Sf\_0107736 | JF825083 |
| LNCR rasiRNA 5 | Sf\_0150035 | JF825084 |
| LNCR rasiRNA 6 | Sf\_0291283 | JF825085 |
| LNCR rasiRNA 7 | Sf\_0065017 | JF825086 |
| LNCR rasiRNA 8 | Sf\_0072866 | JF825087 |
| LNCR rasiRNA 9 | Sf\_0661347 | JF825088 |
| LNCR rasiRNA 10 | Sf\_0611304 | JF825089 |
| LNCR rasiRNA 11 | Sf\_0154566 | JF825090 |
| LNCR rasiRNA 12 | Sf\_0171535 | JF825091 |
| LNCR rasiRNA 13 | Sf\_0109547 | JF825092 |
| LNCR rasiRNA 14 | Sf\_0099316 | JF825093 |
| LNCR rasiRNA 15 | Sf\_0057869 | JF825094 |
| LNCR rasiRNA 16 | Sf\_0105093 | JF825095 |
| LNCR rasiRNA 17 | Sf\_0105102 | JF825096 |
| LNCR rasiRNA 18 | Sf\_0099322 | JF825097 |
| LNCR rasiRNA 19 | Sf\_0285252 | JF825098 |
| LNCR rasiRNA 20 | Sf\_0125293 | JF825099 |
| LNCR rasiRNA 21 | Sf\_0146986 | JF825100 |
| LNCR rasiRNA 22 | Sf\_0175838 | JF825101 |
| LNCR rasiRNA 23 | Sf\_0029718 | JF825102 |
| LNCR rasiRNA 24 | Sf\_0051963 | JF825103 |
| LNCR rasiRNA 25 | Sf\_0093457 | JF825104 |
| LNCR rasiRNA 26 | Sf\_0231851 | JF825105 |
| LNCR rasiRNA 27 | Sf\_0164342 | JF825106 |
| LNCR rasiRNA 28 | Sf\_0158959 | JF825107 |
| LNCR rasiRNA 29 | Sf\_0087454 | JF825108 |
| LNCR rasiRNA 30 | Sf\_0109558 | JF825109 |
| LNCR rasiRNA 31 | Sf\_0171533 | JF825110 |
| LNCR rasiRNA 32 | Sf\_0184632 | JF825111 |
| LNCR rasiRNA 33 | Sf\_0154575 | JF825112 |
| LNCR rasiRNA 34 | Sf\_0291281 | JF825113 |
| LNCR rasiRNA 35 | Sf\_0291302 | JF825114 |
| LNCR rasiRNA 36 | Sf\_0231850 | JF825115 |
| LNCR rasiRNA 37 | Sf\_0483512 | JF825116 |
| LNCR rasiRNA 38 | Sf\_0204233 | JF825117 |
| LNCR rasiRNA 39 | Sf\_0204215 | JF825118 |
| LNCR rasiRNA 40 | Sf\_0237742 | JF825119 |
| LNCR rasiRNA 41 | Sf\_0936377 | JF825120 |
| LNCR rasiRNA 42 | Sf\_1413597 | JF825121 |
| LNCR rasiRNA 43 | Sf\_0177726 | JF825122 |
| LNCR rasiRNA 44 | Sf\_1546380 | JF825123 |
| LNCR rasiRNA 45 | Sf\_1580243 | JF825124 |
| LNCR rasiRNA 46 | Sf\_0605540 | JF825125 |
| Transposable element | TE-LNCR\_copy1 | JF825126 |
| Transposable element | TE-LNCR\_copy2 | JF825127 |
| Transposable element | TE-LNCR\_copy3 | JF825128 |
| Transposable element | TE-LNCR\_copy4 | JF825129 |
| Transposable element | TE-LNCR\_copy5 | JF825130 |
| Transposable element | TE-LNCR\_copy6 | JF825131 |
| Transposable element | TE-LNCR\_copy7 | JF825132 |
| Transposable element | TE-LNCR\_copy8 | JF825133 |
| Transposable element | TE-LNCR\_copy9 | JF825134 |
| Transposable element | TE-LNCR\_copy10 | JF825135 |
| Transposable element | TE-LNCR\_copy11 | JF825136 |
| long non-coding RNA | LNCR | JF825137 |
