# Supplementary material for: Correlation of LNCR rasiRNAs Expression with Heterochromatin Formation during Development of the Holocentric Insect Spodoptera frugiperda
Source: PLoS One. 2011 Sep 30;6(9):e24746. doi: 10.1371/journal.pone.0024746 (PMC3184123; doi:10.1371/journal.pone.0024746)
Supplement: Table S3 — Table of S. frugiperda LNCR rasiRNAs. LNCR rasiRNAs are sorted in cluster 1 and cluster 2 with their names, sequences, percentage in each of three libraries (2.5 days old fertilized eggs, L2 larval stage and 12 days old pupae) and their position on LNCR and the consensus DNA repeated element (TE LNCR consensus or Spodo2-B-R19-Map11_NoCat-consensus (LepidoDB)). (PPTX) [file pone.0024746.s008.pptx]

## Slide 1
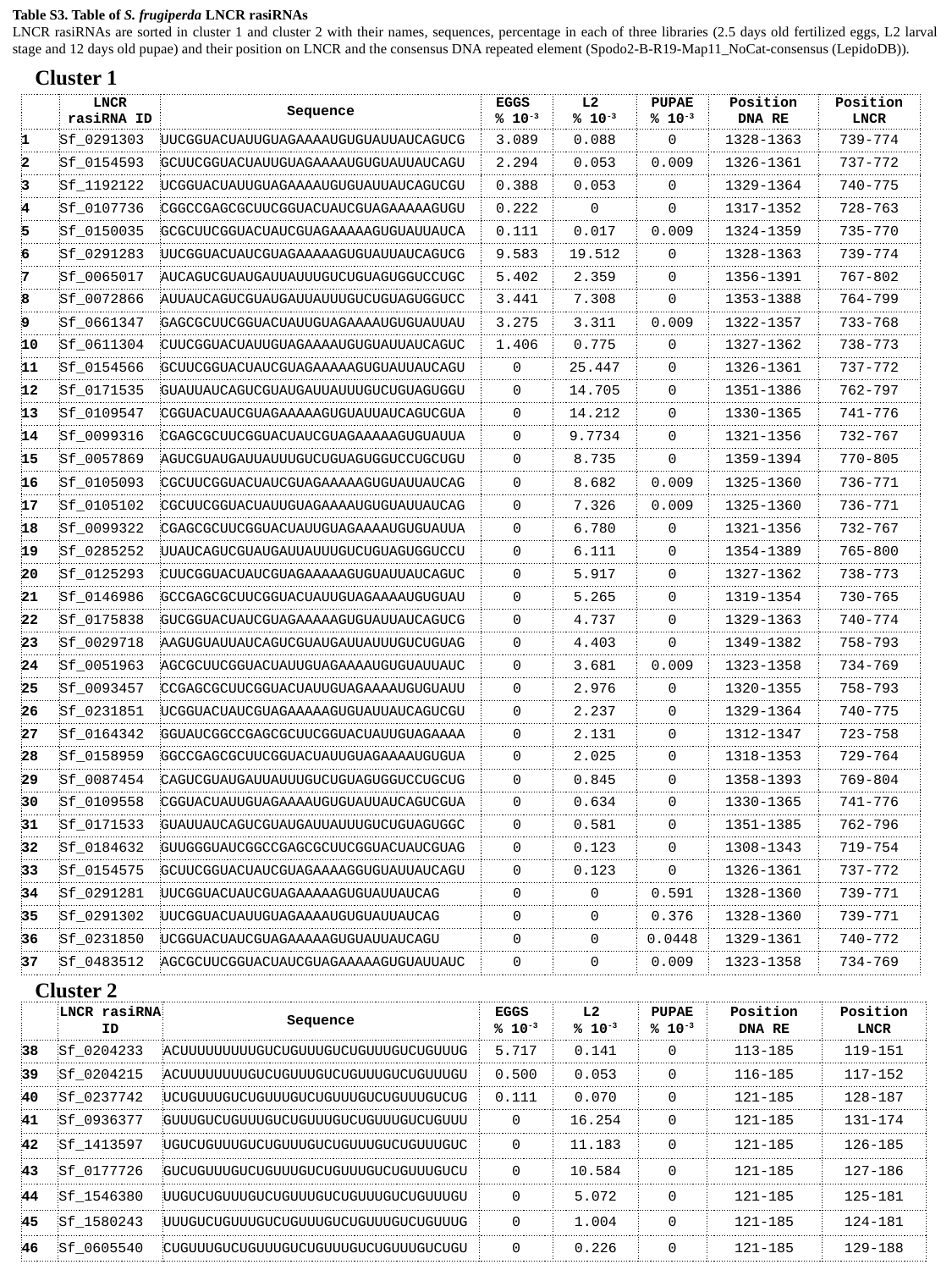

Table S3. Table of S. frugiperda LNCR rasiRNAs
LNCR rasiRNAs are sorted in cluster 1 and cluster 2 with their names, sequences, percentage in each of three libraries (2.5 days old fertilized eggs, L2 larval stage and 12 days old pupae) and their position on LNCR and the consensus DNA repeated element (Spodo2-B-R19-Map11_NoCat-consensus (LepidoDB)).
Cluster 1
| | LNCR rasiRNA ID | Sequence | EGGS % 10-3 | L2 % 10-3 | PUPAE % 10-3 | Position DNA RE | Position LNCR |
| --- | --- | --- | --- | --- | --- | --- | --- |
| 1 | Sf\_0291303 | UUCGGUACUAUUGUAGAAAAUGUGUAUUAUCAGUCG | 3.089 | 0.088 | 0 | 1328-1363 | 739-774 |
| 2 | Sf\_0154593 | GCUUCGGUACUAUUGUAGAAAAUGUGUAUUAUCAGU | 2.294 | 0.053 | 0.009 | 1326-1361 | 737-772 |
| 3 | Sf\_1192122 | UCGGUACUAUUGUAGAAAAUGUGUAUUAUCAGUCGU | 0.388 | 0.053 | 0 | 1329-1364 | 740-775 |
| 4 | Sf\_0107736 | CGGCCGAGCGCUUCGGUACUAUCGUAGAAAAAGUGU | 0.222 | 0 | 0 | 1317-1352 | 728-763 |
| 5 | Sf\_0150035 | GCGCUUCGGUACUAUCGUAGAAAAAGUGUAUUAUCA | 0.111 | 0.017 | 0.009 | 1324-1359 | 735-770 |
| 6 | Sf\_0291283 | UUCGGUACUAUCGUAGAAAAAGUGUAUUAUCAGUCG | 9.583 | 19.512 | 0 | 1328-1363 | 739-774 |
| 7 | Sf\_0065017 | AUCAGUCGUAUGAUUAUUUGUCUGUAGUGGUCCUGC | 5.402 | 2.359 | 0 | 1356-1391 | 767-802 |
| 8 | Sf\_0072866 | AUUAUCAGUCGUAUGAUUAUUUGUCUGUAGUGGUCC | 3.441 | 7.308 | 0 | 1353-1388 | 764-799 |
| 9 | Sf\_0661347 | GAGCGCUUCGGUACUAUUGUAGAAAAUGUGUAUUAU | 3.275 | 3.311 | 0.009 | 1322-1357 | 733-768 |
| 10 | Sf\_0611304 | CUUCGGUACUAUUGUAGAAAAUGUGUAUUAUCAGUC | 1.406 | 0.775 | 0 | 1327-1362 | 738-773 |
| 11 | Sf\_0154566 | GCUUCGGUACUAUCGUAGAAAAAGUGUAUUAUCAGU | 0 | 25.447 | 0 | 1326-1361 | 737-772 |
| 12 | Sf\_0171535 | GUAUUAUCAGUCGUAUGAUUAUUUGUCUGUAGUGGU | 0 | 14.705 | 0 | 1351-1386 | 762-797 |
| 13 | Sf\_0109547 | CGGUACUAUCGUAGAAAAAGUGUAUUAUCAGUCGUA | 0 | 14.212 | 0 | 1330-1365 | 741-776 |
| 14 | Sf\_0099316 | CGAGCGCUUCGGUACUAUCGUAGAAAAAGUGUAUUA | 0 | 9.7734 | 0 | 1321-1356 | 732-767 |
| 15 | Sf\_0057869 | AGUCGUAUGAUUAUUUGUCUGUAGUGGUCCUGCUGU | 0 | 8.735 | 0 | 1359-1394 | 770-805 |
| 16 | Sf\_0105093 | CGCUUCGGUACUAUCGUAGAAAAAGUGUAUUAUCAG | 0 | 8.682 | 0.009 | 1325-1360 | 736-771 |
| 17 | Sf\_0105102 | CGCUUCGGUACUAUUGUAGAAAAUGUGUAUUAUCAG | 0 | 7.326 | 0.009 | 1325-1360 | 736-771 |
| 18 | Sf\_0099322 | CGAGCGCUUCGGUACUAUUGUAGAAAAUGUGUAUUA | 0 | 6.780 | 0 | 1321-1356 | 732-767 |
| 19 | Sf\_0285252 | UUAUCAGUCGUAUGAUUAUUUGUCUGUAGUGGUCCU | 0 | 6.111 | 0 | 1354-1389 | 765-800 |
| 20 | Sf\_0125293 | CUUCGGUACUAUCGUAGAAAAAGUGUAUUAUCAGUC | 0 | 5.917 | 0 | 1327-1362 | 738-773 |
| 21 | Sf\_0146986 | GCCGAGCGCUUCGGUACUAUUGUAGAAAAUGUGUAU | 0 | 5.265 | 0 | 1319-1354 | 730-765 |
| 22 | Sf\_0175838 | GUCGGUACUAUCGUAGAAAAAGUGUAUUAUCAGUCG | 0 | 4.737 | 0 | 1329-1363 | 740-774 |
| 23 | Sf\_0029718 | AAGUGUAUUAUCAGUCGUAUGAUUAUUUGUCUGUAG | 0 | 4.403 | 0 | 1349-1382 | 758-793 |
| 24 | Sf\_0051963 | AGCGCUUCGGUACUAUUGUAGAAAAUGUGUAUUAUC | 0 | 3.681 | 0.009 | 1323-1358 | 734-769 |
| 25 | Sf\_0093457 | CCGAGCGCUUCGGUACUAUUGUAGAAAAUGUGUAUU | 0 | 2.976 | 0 | 1320-1355 | 758-793 |
| 26 | Sf\_0231851 | UCGGUACUAUCGUAGAAAAAGUGUAUUAUCAGUCGU | 0 | 2.237 | 0 | 1329-1364 | 740-775 |
| 27 | Sf\_0164342 | GGUAUCGGCCGAGCGCUUCGGUACUAUUGUAGAAAA | 0 | 2.131 | 0 | 1312-1347 | 723-758 |
| 28 | Sf\_0158959 | GGCCGAGCGCUUCGGUACUAUUGUAGAAAAUGUGUA | 0 | 2.025 | 0 | 1318-1353 | 729-764 |
| 29 | Sf\_0087454 | CAGUCGUAUGAUUAUUUGUCUGUAGUGGUCCUGCUG | 0 | 0.845 | 0 | 1358-1393 | 769-804 |
| 30 | Sf\_0109558 | CGGUACUAUUGUAGAAAAUGUGUAUUAUCAGUCGUA | 0 | 0.634 | 0 | 1330-1365 | 741-776 |
| 31 | Sf\_0171533 | GUAUUAUCAGUCGUAUGAUUAUUUGUCUGUAGUGGC | 0 | 0.581 | 0 | 1351-1385 | 762-796 |
| 32 | Sf\_0184632 | GUUGGGUAUCGGCCGAGCGCUUCGGUACUAUCGUAG | 0 | 0.123 | 0 | 1308-1343 | 719-754 |
| 33 | Sf\_0154575 | GCUUCGGUACUAUCGUAGAAAAGGUGUAUUAUCAGU | 0 | 0.123 | 0 | 1326-1361 | 737-772 |
| 34 | Sf\_0291281 | UUCGGUACUAUCGUAGAAAAAGUGUAUUAUCAG | 0 | 0 | 0.591 | 1328-1360 | 739-771 |
| 35 | Sf\_0291302 | UUCGGUACUAUUGUAGAAAAUGUGUAUUAUCAG | 0 | 0 | 0.376 | 1328-1360 | 739-771 |
| 36 | Sf\_0231850 | UCGGUACUAUCGUAGAAAAAGUGUAUUAUCAGU | 0 | 0 | 0.0448 | 1329-1361 | 740-772 |
| 37 | Sf\_0483512 | AGCGCUUCGGUACUAUCGUAGAAAAAGUGUAUUAUC | 0 | 0 | 0.009 | 1323-1358 | 734-769 |
Cluster 2
| | LNCR rasiRNA ID | Sequence | EGGS % 10-3 | L2 % 10-3 | PUPAE % 10-3 | Position DNA RE | Position LNCR |
| --- | --- | --- | --- | --- | --- | --- | --- |
| 38 | Sf\_0204233 | ACUUUUUUUUUGUCUGUUUGUCUGUUUGUCUGUUUG | 5.717 | 0.141 | 0 | 113-185 | 119-151 |
| 39 | Sf\_0204215 | ACUUUUUUUUGUCUGUUUGUCUGUUUGUCUGUUUGU | 0.500 | 0.053 | 0 | 116-185 | 117-152 |
| 40 | Sf\_0237742 | UCUGUUUGUCUGUUUGUCUGUUUGUCUGUUUGUCUG | 0.111 | 0.070 | 0 | 121-185 | 128-187 |
| 41 | Sf\_0936377 | GUUUGUCUGUUUGUCUGUUUGUCUGUUUGUCUGUUU | 0 | 16.254 | 0 | 121-185 | 131-174 |
| 42 | Sf\_1413597 | UGUCUGUUUGUCUGUUUGUCUGUUUGUCUGUUUGUC | 0 | 11.183 | 0 | 121-185 | 126-185 |
| 43 | Sf\_0177726 | GUCUGUUUGUCUGUUUGUCUGUUUGUCUGUUUGUCU | 0 | 10.584 | 0 | 121-185 | 127-186 |
| 44 | Sf\_1546380 | UUGUCUGUUUGUCUGUUUGUCUGUUUGUCUGUUUGU | 0 | 5.072 | 0 | 121-185 | 125-181 |
| 45 | Sf\_1580243 | UUUGUCUGUUUGUCUGUUUGUCUGUUUGUCUGUUUG | 0 | 1.004 | 0 | 121-185 | 124-181 |
| 46 | Sf\_0605540 | CUGUUUGUCUGUUUGUCUGUUUGUCUGUUUGUCUGU | 0 | 0.226 | 0 | 121-185 | 129-188 |
