# Supplementary material for: Correlation of LNCR rasiRNAs Expression with Heterochromatin Formation during Development of the Holocentric Insect Spodoptera frugiperda
Source: PLoS One. 2011 Sep 30;6(9):e24746. doi: 10.1371/journal.pone.0024746 (PMC3184123; doi:10.1371/journal.pone.0024746)
Supplement: Table S4 — Mapping of LNCR rasiRNAs to genomic regions. Position of the LNCR rasiRNAs on some of the S. frugiperda genomic regions that are cloned in bacterial artificial chromosome (BAC) and sequenced. Only perfect matches are presented. (PPTX) [file pone.0024746.s009.pptx]

## Slide 1
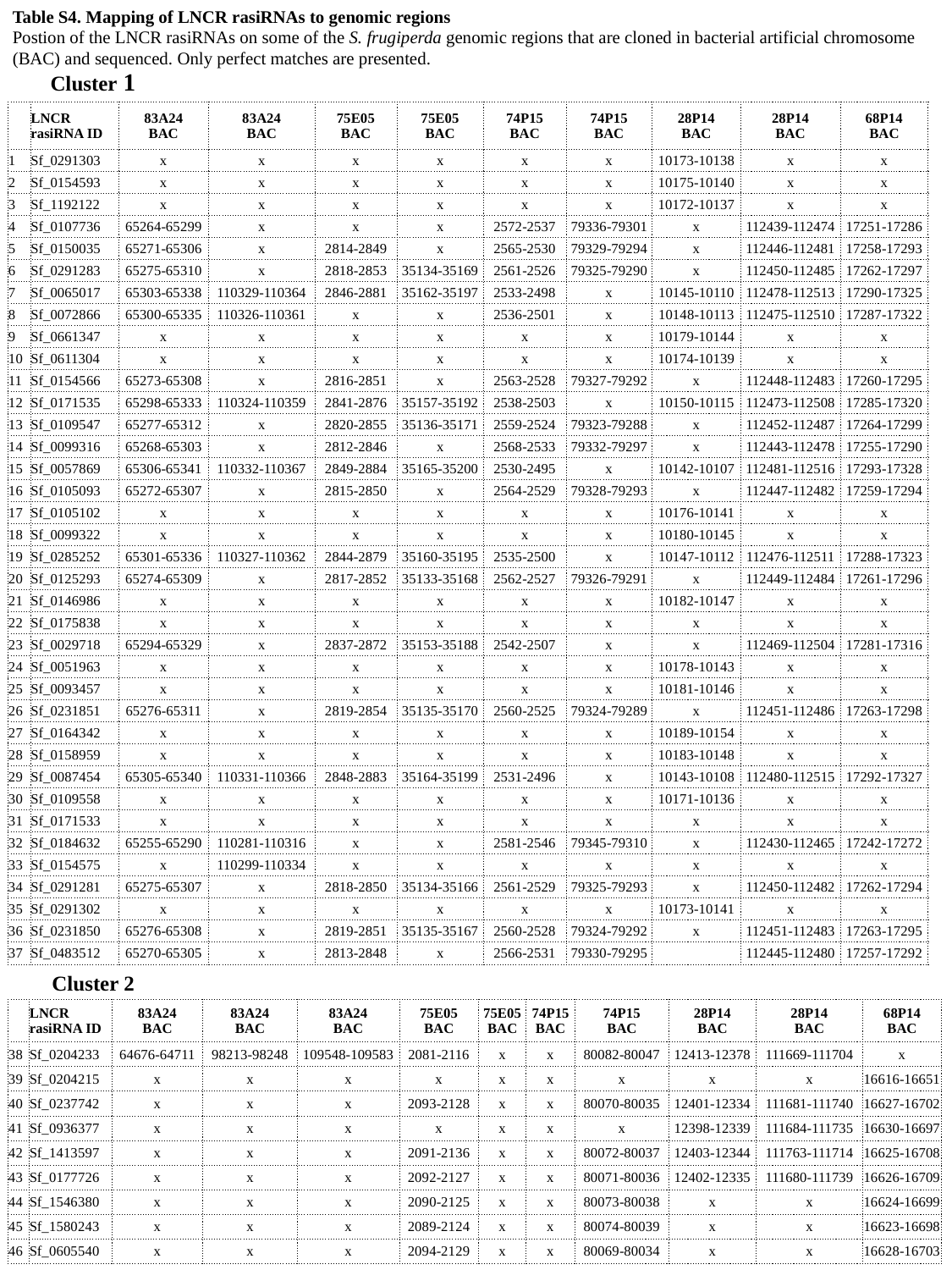

Table S4. Mapping of LNCR rasiRNAs to genomic regions
Postion of the LNCR rasiRNAs on some of the S. frugiperda genomic regions that are cloned in bacterial artificial chromosome (BAC) and sequenced. Only perfect matches are presented.
Cluster 1
| | LNCR rasiRNA ID | 83A24 BAC | 83A24 BAC | 75E05 BAC | 75E05 BAC | 74P15 BAC | 74P15 BAC | 28P14 BAC | 28P14 BAC | 68P14 BAC |
| --- | --- | --- | --- | --- | --- | --- | --- | --- | --- | --- |
| 1 | Sf\_0291303 | x | x | x | x | x | x | 10173-10138 | x | x |
| 2 | Sf\_0154593 | x | x | x | x | x | x | 10175-10140 | x | x |
| 3 | Sf\_1192122 | x | x | x | x | x | x | 10172-10137 | x | x |
| 4 | Sf\_0107736 | 65264-65299 | x | x | x | 2572-2537 | 79336-79301 | x | 112439-112474 | 17251-17286 |
| 5 | Sf\_0150035 | 65271-65306 | x | 2814-2849 | x | 2565-2530 | 79329-79294 | x | 112446-112481 | 17258-17293 |
| 6 | Sf\_0291283 | 65275-65310 | x | 2818-2853 | 35134-35169 | 2561-2526 | 79325-79290 | x | 112450-112485 | 17262-17297 |
| 7 | Sf\_0065017 | 65303-65338 | 110329-110364 | 2846-2881 | 35162-35197 | 2533-2498 | x | 10145-10110 | 112478-112513 | 17290-17325 |
| 8 | Sf\_0072866 | 65300-65335 | 110326-110361 | x | x | 2536-2501 | x | 10148-10113 | 112475-112510 | 17287-17322 |
| 9 | Sf\_0661347 | x | x | x | x | x | x | 10179-10144 | x | x |
| 10 | Sf\_0611304 | x | x | x | x | x | x | 10174-10139 | x | x |
| 11 | Sf\_0154566 | 65273-65308 | x | 2816-2851 | x | 2563-2528 | 79327-79292 | x | 112448-112483 | 17260-17295 |
| 12 | Sf\_0171535 | 65298-65333 | 110324-110359 | 2841-2876 | 35157-35192 | 2538-2503 | x | 10150-10115 | 112473-112508 | 17285-17320 |
| 13 | Sf\_0109547 | 65277-65312 | x | 2820-2855 | 35136-35171 | 2559-2524 | 79323-79288 | x | 112452-112487 | 17264-17299 |
| 14 | Sf\_0099316 | 65268-65303 | x | 2812-2846 | x | 2568-2533 | 79332-79297 | x | 112443-112478 | 17255-17290 |
| 15 | Sf\_0057869 | 65306-65341 | 110332-110367 | 2849-2884 | 35165-35200 | 2530-2495 | x | 10142-10107 | 112481-112516 | 17293-17328 |
| 16 | Sf\_0105093 | 65272-65307 | x | 2815-2850 | x | 2564-2529 | 79328-79293 | x | 112447-112482 | 17259-17294 |
| 17 | Sf\_0105102 | x | x | x | x | x | x | 10176-10141 | x | x |
| 18 | Sf\_0099322 | x | x | x | x | x | x | 10180-10145 | x | x |
| 19 | Sf\_0285252 | 65301-65336 | 110327-110362 | 2844-2879 | 35160-35195 | 2535-2500 | x | 10147-10112 | 112476-112511 | 17288-17323 |
| 20 | Sf\_0125293 | 65274-65309 | x | 2817-2852 | 35133-35168 | 2562-2527 | 79326-79291 | x | 112449-112484 | 17261-17296 |
| 21 | Sf\_0146986 | x | x | x | x | x | x | 10182-10147 | x | x |
| 22 | Sf\_0175838 | x | x | x | x | x | x | x | x | x |
| 23 | Sf\_0029718 | 65294-65329 | x | 2837-2872 | 35153-35188 | 2542-2507 | x | x | 112469-112504 | 17281-17316 |
| 24 | Sf\_0051963 | x | x | x | x | x | x | 10178-10143 | x | x |
| 25 | Sf\_0093457 | x | x | x | x | x | x | 10181-10146 | x | x |
| 26 | Sf\_0231851 | 65276-65311 | x | 2819-2854 | 35135-35170 | 2560-2525 | 79324-79289 | x | 112451-112486 | 17263-17298 |
| 27 | Sf\_0164342 | x | x | x | x | x | x | 10189-10154 | x | x |
| 28 | Sf\_0158959 | x | x | x | x | x | x | 10183-10148 | x | x |
| 29 | Sf\_0087454 | 65305-65340 | 110331-110366 | 2848-2883 | 35164-35199 | 2531-2496 | x | 10143-10108 | 112480-112515 | 17292-17327 |
| 30 | Sf\_0109558 | x | x | x | x | x | x | 10171-10136 | x | x |
| 31 | Sf\_0171533 | x | x | x | x | x | x | x | x | x |
| 32 | Sf\_0184632 | 65255-65290 | 110281-110316 | x | x | 2581-2546 | 79345-79310 | x | 112430-112465 | 17242-17272 |
| 33 | Sf\_0154575 | x | 110299-110334 | x | x | x | x | x | x | x |
| 34 | Sf\_0291281 | 65275-65307 | x | 2818-2850 | 35134-35166 | 2561-2529 | 79325-79293 | x | 112450-112482 | 17262-17294 |
| 35 | Sf\_0291302 | x | x | x | x | x | x | 10173-10141 | x | x |
| 36 | Sf\_0231850 | 65276-65308 | x | 2819-2851 | 35135-35167 | 2560-2528 | 79324-79292 | x | 112451-112483 | 17263-17295 |
| 37 | Sf\_0483512 | 65270-65305 | x | 2813-2848 | x | 2566-2531 | 79330-79295 | | 112445-112480 | 17257-17292 |
Cluster 2
| | LNCR rasiRNA ID | 83A24 BAC | 83A24 BAC | 83A24 BAC | 75E05 BAC | 75E05 BAC | 74P15 BAC | 74P15 BAC | 28P14 BAC | 28P14 BAC | 68P14 BAC |
| --- | --- | --- | --- | --- | --- | --- | --- | --- | --- | --- | --- |
| 38 | Sf\_0204233 | 64676-64711 | 98213-98248 | 109548-109583 | 2081-2116 | x | x | 80082-80047 | 12413-12378 | 111669-111704 | x |
| 39 | Sf\_0204215 | x | x | x | x | x | x | x | x | x | 16616-16651 |
| 40 | Sf\_0237742 | x | x | x | 2093-2128 | x | x | 80070-80035 | 12401-12334 | 111681-111740 | 16627-16702 |
| 41 | Sf\_0936377 | x | x | x | x | x | x | x | 12398-12339 | 111684-111735 | 16630-16697 |
| 42 | Sf\_1413597 | x | x | x | 2091-2136 | x | x | 80072-80037 | 12403-12344 | 111763-111714 | 16625-16708 |
| 43 | Sf\_0177726 | x | x | x | 2092-2127 | x | x | 80071-80036 | 12402-12335 | 111680-111739 | 16626-16709 |
| 44 | Sf\_1546380 | x | x | x | 2090-2125 | x | x | 80073-80038 | x | x | 16624-16699 |
| 45 | Sf\_1580243 | x | x | x | 2089-2124 | x | x | 80074-80039 | x | x | 16623-16698 |
| 46 | Sf\_0605540 | x | x | x | 2094-2129 | x | x | 80069-80034 | x | x | 16628-16703 |
